# Supplementary material for: INTEGRATE: Model-based multi-omics data integration to characterize multi-level metabolic regulation
Source: PLoS Comput Biol. 2022 Feb 7;18(2):e1009337. doi: 10.1371/journal.pcbi.1009337 (PMC8853556; doi:10.1371/journal.pcbi.1009337)
Supplement: S1 Fig — Graphical explanation of transcriptional, metabolic and combined metabolic and transcriptional control. (PDF) [file pcbi.1009337.s001.pdf]

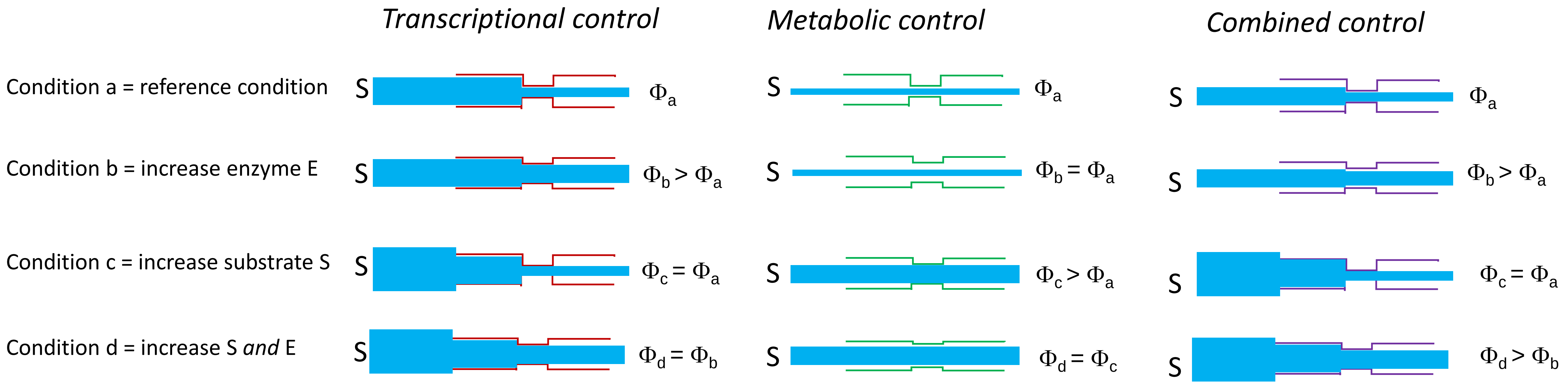
Transcriptional control
Metabolic control
Combined control
Condition a = reference condition
Condition b = increase enzyme E
Condition c = increase substrate S
Condition d = increase S *and* E
S
Φ_a
$\Phi_b > \Phi_a$
$\Phi_c = \Phi_a$
$\Phi_d = \Phi_b$
Φ_a
$\Phi_b = \Phi_a$
$\Phi_c > \Phi_a$
$\Phi_d = \Phi_c$
Φ_a
$\Phi_b > \Phi_a$
$\Phi_c = \Phi_a$
$\Phi_d > \Phi_b$
