# Supplementary figures and images for: INTEGRATE: Model-based multi-omics data integration to characterize multi-level metabolic regulation
Source: PLoS Comput Biol. 2022 Feb 7;18(2):e1009337. doi: 10.1371/journal.pcbi.1009337 (PMC8853556; doi:10.1371/journal.pcbi.1009337)

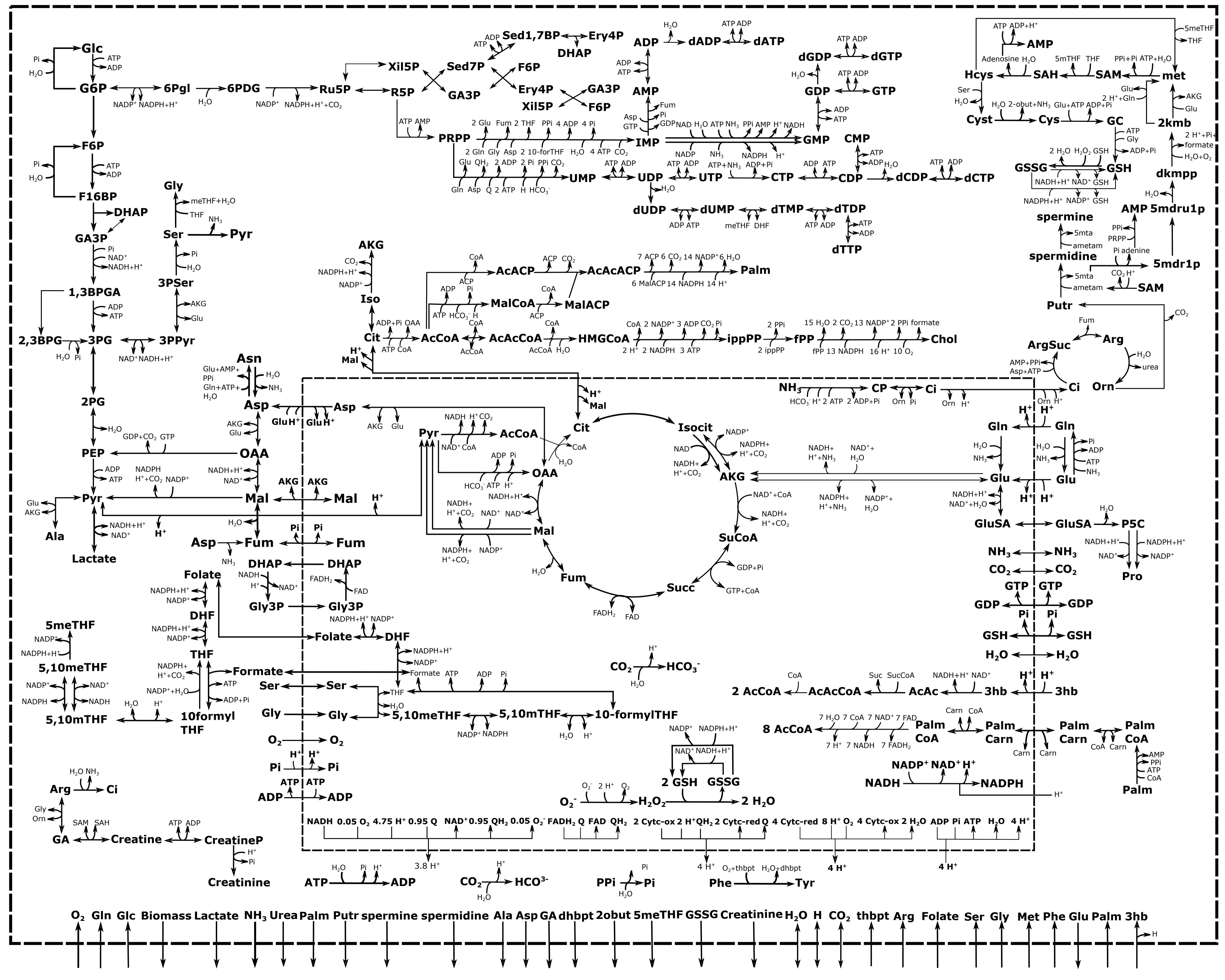

Supplement: S2 Fig — Graphical representations of central carbon metabolism reactions included in ENGRO2 model. (PDF) [file pcbi.1009337.s002.pdf]

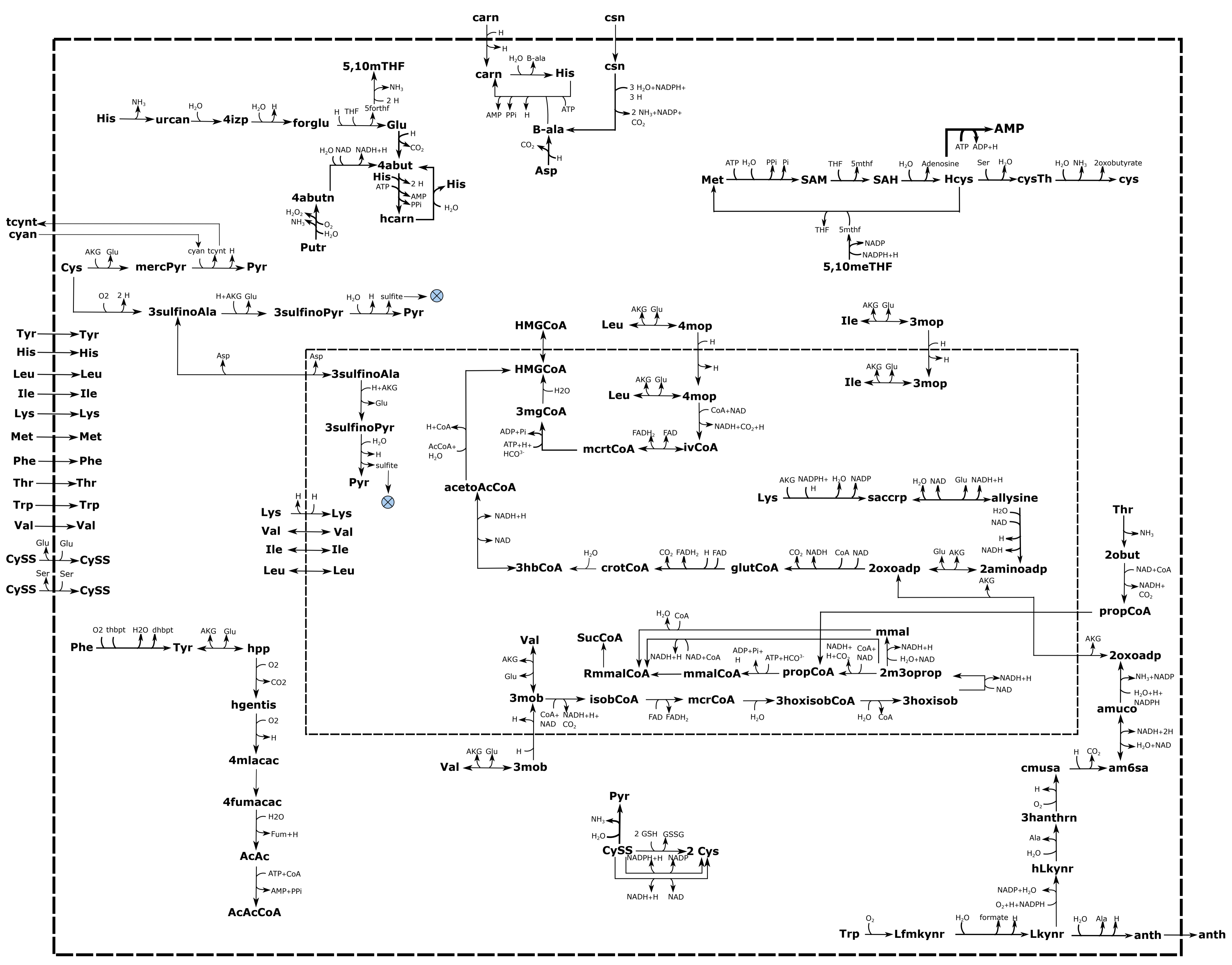

Supplement: S3 Fig — Graphical representations of essential amino acid metabolism reactions included in ENGRO2 model. (PDF) [file pcbi.1009337.s003.pdf]
