# Supplementary material for: INTEGRATE: Model-based multi-omics data integration to characterize multi-level metabolic regulation
Source: PLoS Comput Biol. 2022 Feb 7;18(2):e1009337. doi: 10.1371/journal.pcbi.1009337 (PMC8853556; doi:10.1371/journal.pcbi.1009337)
Supplement: S4 Fig — Diagram recapitulating all the INTEGRATE steps for a specific toy reaction. (PDF) [file pcbi.1009337.s004.pdf]

1 >> for a given model reaction R

Formula: 2A + B => C

GPR: G1 AND G2

2 >> compute RPS of R for each sample

Metabolite abundances

Metabolite	MCF7_1	MCF7_2	…
A	5	5	6
B	10	11	10
…			

$RPS = [A]^2[B]$

Reaction	MCF7_1	MCF7_2	…
R	$5^2*10=250$	$5^2*11=275$	$6^2*10=360$

3 >> compute RAS of R for each sample

Read counts

Gene	MCF7_1	MCF7_2	…
G1	0	1	0
G2	10	11	10
…			

RAS = min (G1,G2)

Reaction	MCF7_1	MCF7_2	…
R	Min(0,10)=0	Min(1,11)=1	Min(0,10) =0

4 >> repeat step 3 for each model reaction and use the RASs to constrain flux boundaries and get a sample of FFDs for reaction R

5 >> comparison between the samples associated with each pair of cell lines, for reaction R

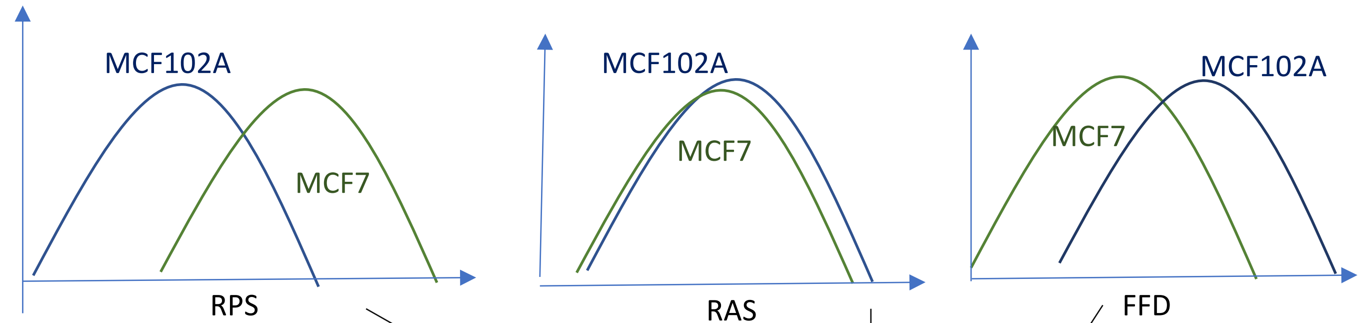

RPS

T-test =1 and fold-change ≧1.2

RAS

T-test = 0 and 0.8 ≦ fold-change ≦ 1.2

FFD

Mann-Withney U test =-1 and fold-change ≦ 0.8

Pair	RPS	RAS	FFD
MCF102A vs MCF7	1	0	-1
MCF102A vs MDAMB231	1	-1	1
MCF102A vs MDAMB361	0	-1	0
….	….	….	….

6>> concordance Analysis for reaction R

Contingency table FFD vs RPS

FFD / RPS	-1	0	1
-1	2	0	0
0	0	3	0
1	0	0	5

→ Cohen's kappa Coefficient: 1

Contingency table RPS vs RAS

RAS / RPS	-1.0	1.0
-1	0	2
0	1	2
1	1	4

→ Cohen's kappa Coefficient: -0.098
