# Supplementary material for: INTEGRATE: Model-based multi-omics data integration to characterize multi-level metabolic regulation
Source: PLoS Comput Biol. 2022 Feb 7;18(2):e1009337. doi: 10.1371/journal.pcbi.1009337 (PMC8853556; doi:10.1371/journal.pcbi.1009337)
Supplement: Attachment — Submitted filename: review_comments.pdf [file pcbi.1009337.s011.pdf]

Review summary

The submitted manuscript by Di Filippo et al. integrates transcriptome and metabolome data with stoichiometric models to unravel whether specific metabolic fluxes are governed by transcriptional regulation, differences in substrate levels, or both. The authors apply their method to study a set of cancer cell lines that have a clear difference in growth rates, substrate uptake and secretion of glutamate and lactate, intracellular metabolite concentrations and gene expression levels. Their main finding is a description of how the two different modes of flux control affects a set of specific reactions in the core metabolism of cancer cells. The study includes further development of a core metabolic model for cancer cell lines with about 500 reactions and metabolites.

The proposed method (INTEGRATE) uses relative gene expression levels (translated to "reaction activity scores (RAS)" through GPR associations) to scale metabolic fluxes in each cancer cell line relative to the maximum flux identified using flux variability analysis (linearly). Then, they apply constraints on exchange fluxes according to measure substrate consumption and secretion of lactate and glutamate (as relative yields), and other differences metabolite availability as dictated by the different cultivation media used for the different cell lines. The authors then sample the solution spaces for the different models (corresponding to the different cell lines) to obtain "feasible flux distributions" (FFD). In parallel, the authors compute "reaction propensity scores (RPS)" for each reaction in each model (the product of substrate concentrations). The authors then perform pairwise comparisons of FFD, RAS and RPS scores for each reaction to investigate whether these values are significantly different between each pair. These scores are then used to calculate the agreement (or concordance as it is called in the manuscript) between FFD and RPS, and between RAS and RPS.

The underlying hypothesis is that the agreement (concordance) of FFD-RPS and RAS-RPS can be used to distinguish whether a reaction is controlled by transcriptional regulation or by substrate levels. I find the hypothesis interesting and valuable and as such, I think the manuscript provides value to the COBRA community. However, my main concern is that the manuscript does not validate the method, and it is therefore yet to be seen whether the main hypothesis is true and if the proposed method can provide valuable predictions. Although lack of proper experimental validation is (unfortunately) not uncommon in this field of research, I'm particularly concerned about experimental validation when transcriptome data is used to infer reaction fluxes (see review by Machado and Herrgård, PLOS Comp. Biol, 2014). Furthermore, and as the authors point out themselves, the underlying assumptions (linear relation between substrate concentrations and flux and linear relation between transcription (RAS) and flux) are not always valid, and hence, it suggests that some validation is needed to determine the usefulness of the proposed method.

Major concerns

- The github repository (https://github.com/qLSLab/integrate) is not publicly available, and it is therefore impossible to evaluate the associated code
- The authors use a designated core model. It would be very valuable to understand how sensitive the proposed method is to the model structure. This could potentially be tested by either making a set of core models integrating different aspects of a full recon3D / HMR model, or by applying their method to Recon3D, HMR, or HMR core model (possibly contextualized using the transcriptome-data) and observe if the method infers similar regulatory mechanisms. Similarly, application of the method to an additional species/context could display generic applicability of the method.

- According to the diagram in Fig. 1 there are three potential regulatory options: metabolic, transcriptional and both. However, as the method does not account for allosteric regulation, product inhibition, or cofactors / prosthetic groups, one would anticipate that one could not pinpoint the regulatory mechanism for all reactions, i.e. should it be an "other" category in this diagram? Furthermore, the upper left quadrant is not explicitly mentioned, but there are reactions in this quadrant in Fig 4A. Can the authors elaborate on how one should interpret these results?
- Although the methods used to generate results in Fig. 5 and 6 are elegant, I think the last part of the results and discussion could be shortened. I think it would be better to clearly summarize / show / verify the proposed method rather than exploring other metabolic differences between the cancer cell lines in such detail. Maybe Fig. 5 and 6. Could be combined into one, as a short proof-of-concept figure summarizing the metabolic differences between the cell lines?
- On page 17, line 425 the authors write "Remarkably, we identified reactions for which there is a good agreement between flux variations and variation in RPSs". If the metabolomic data or transcriptome data was completely random, how many reactions with an agreement between RPSs and flux variations is expected just by chance?
- How do you ensure that the flux sampling don't provide unrealistic flux values caused by loops when you split all reversible reactions into two irreversible?
- Figure 3B: It seems to be a difference between the computational and experimental yields on the scale of two orders of magnitude. Why?

Minor concerns and comments:

- Abstract: It seems more logical to present transcriptional regulation as the first class of regulation and regulation as the second class, and I think it will be easier to read if presented in this order.
- Figure 2D. Color scale could be diverging from 0 to better display above and below average. The meaning of the colorbar-legend is also somewhat unclear, as abundances can't be negative. Is it Z-scores?
- Figure 2F. I think it would be easier to interpret a normal semilog (log10) Y-axis scale. Also, error bars are difficult to see.
- Discussion: Can you discuss whether the current framework can be extended to also cover allosteric and cofactor regulation?
- Discussion: Can proteomics be used rather than transcriptomics to predict RAS scores? Would the authors anticipate better results from proteomics rather than transcriptomics?
- Author summary: proteomics can also give clues on metabolism, not only metabolomics and transcriptomics
- Line 42: typo, transcriptional control
- Line 42-50: Rewrite for clarity. I suggest considering the flux through the reaction r1 rather than enzyme E1. Last bullet point should say something along the lines: "flux through r1 is co-regulated with changes in [s1] and [E1].
- Line 67: Suggest citing some of the seminal papers or reviews on integration of GEMs and omics data her.
- Line 71: Unsteady FBA should also be cited here.

- FVA: Using 1e-4 as a lower bound for growth will result in somewhat unrealistic flux boundaries. If you use a value closer to the measured growth rates I expect that you would get more realistic flux bounds. How does this affect downstream analysis?
- Line 107-108: combine into one paragraph.
- For how many reactions in the models were you able to calculate a RPS?
- Line 145: Typo and rewrite: E.g. Reactions … high FFD-RPS are under metabolic control.
- Figure 4A: Should the figure display a boundary at 0.2 concordance, as this is considered poor concordance? Also, write out proper x- and y-axis labels.
- Figure 4B: Maybe have a separate column with reaction name / id to make it easier to read.
- Figure 4C: Not really useful.
- Figure 5B/C: Mention that color of names corresponds to flux distributions to the right.
- Figure 6: Although exchange fluxes are indeed negative in GEMs when substrates are consumed, it might be easier to interpret this result if positive values are used, labelled substrate uptake. Furthermore, the y-axis label seems strange; assuming that the biomass coefficients is fixed it is a constant relative abundance of each specific amino acid. Isn't the y-axis here basically the flux drain to growth, also in mmol/gDW/h?
- Figure 5B/C: Fluxes seems unrealistically high
