# Supplementary material for: INTEGRATE: Model-based multi-omics data integration to characterize multi-level metabolic regulation
Source: PLoS Comput Biol. 2022 Feb 7;18(2):e1009337. doi: 10.1371/journal.pcbi.1009337 (PMC8853556; doi:10.1371/journal.pcbi.1009337)
Supplement: Attachment — Submitted filename: Review_PCOMPBIOL-D-21-01369.pdf [file pcbi.1009337.s012.pdf]

Materials and Methods:
I like to start reading this part of a manuscript to evaluate, whether I can understand the experiment and line of though based on the abstract. Further, I want to evaluate, whether I could reproduce the experiments based on the descriptions.

General note: The authors are inconsistent: For example, in line 524 the "*S* matrix", and in 528 "matrix S". In the experimental part, where *µ*L is in italics, whereas all other units are not (nM, mL).
The authors should also proof-read the whole section, as e.g. line 736 mentions "at least 8 x 106 cells" and line 696 "CO2", which are easy errors to spot.

Model reconstruction:

1. Which program was used, which version, functions to be cited,…?
2. Which model was actually used? ENGRO2 or 1, or construct a new one based on both? Are these published models? (as there is no citations) In the supplementary is the ENGRO2 model listed, does that include the changes/integrations made in this manuscript? Can the authors please clarify which model the authors are discussing about, e.g. name the model used in the manuscript ENGRO2_met_tra for easier distinction.
3. Based on the text in the chapter "Model reconstruction", I would not be able to reconstruct the model. E.g. "it was necessary to add the ROS detoxification pathway by means of glutathione" or "we enriched the ENGRO2 model by including both folate and the methionine cycles" does not tell me anything about actual changes to the model.
4. How does the Biomass reaction look like based on the last-paragraph description? I assume, the authors removed the mentioned compounds, added the molar mass equivalent to the biomass and rescaled the whole biomass function?

FVA:

1. FVA was not developed by Gudmundsson and Thiele.
2. The one sentence summary is hard to understand, something along the lines of "FVA determines the maximal (and minimal) flux possible through any reaction of the model and thus evaluates the cell's range of metabolic capabilities."
3. The statement "As other constraint based modelling methods, it relies on the fact that the steady states of the metabolic model corresponds to the kernel of the stoichiometric matrix S associated to the model itself" should be supported by citations. The next sentence reads "It is exactly this null space that defines the space of all the feasible fluxes.", which makes me wonder about the null space, as this is not mentioned before. (This is complaining on a high level though, I apologize for that)
4. I do not understand the following sentence: "Moreover, to mimic as closely as possible the biological process in analysis, it is possible to bound the feasible fluxes space by means of convex half planes represented by the reaction's lower and upper bounds." I think, here is some English-language fixing necessary, and a citation.
5. Since this is "materials and methods": The method was FVA, but what did the authors do with the FVA? And how? In which program?
6. I am not sure, whether the usage of FVA is correctly in this manuscript. Usually (as the authors state) one would use FVA to determine the variable fluxes given a percentage flux trough the objective function. Relaxing that percentage will result in broader variable flux ranges, as all reaction fluxes can be redistributed fulfilling the objective flux requirement. The authors state

here that they only want to prevent a non-zero objective function flux. This should relax all other fluxes to their absolute maximum, so basically, determine what the network restrictions are in a given environment. Would this actually constrain the model fluxes (sure, it might constrain the lb/ub of a given reaction instead of -1000/1000, but they would be constraint by the medium to the same values, right?)

Reaction activity Score computation

1. GPR rule description (line 542++) Please add a citation for the mentioned GPR rules. From microbe models, the GPR rules are for genes, where multiple (linked by these rules) can encode for one reaction (protein). AND means then that both genes are needed (in case of knock out relevant), OR means that either is needed. Please include a citation for GPR rules on an enzyme level, also encoding for multimeters.
2. "According to [31]" → Please incorporate a one to two line summary of the method. Please also formulate how the RAS is (mathematically as equation) defined.
3. Line 560 "in all the cell line" → add the plural "cell lines"
4. "consider that the corresponding reaction is off in all the cell lines" reformulate, maybe "inactive" instead of "off"?

Constraints on nutrient availability

1. The description of uptake- and secretion fluxes is great. However, this section (in materials and methods) does not provide the reader with any information about the medium used for the models, (reactions), nor any of the uptake fluxes.

Constraints on extracellular fluxes

2. Reading on, I understand that the previous section is an introduction for this one, with a different headline?
3. The authors use the ratios of three pairs of metabolites to determine exchange rates. What were all the other exchange rates? Zero?
4. To clarify: The authors use two pints (zero and 48 hours) to determine uptake rates (which is way too little data points, hence the authors use the ratios?). The authors did measure two concentrations (for a given metabolite at both time points), and calculated the rate… how? This needs information about the number of cells and cell dry weight. I assume, I'll read about these measurements later, however a link to the later methods part would be nice.
5. Also: These calculated data as well as the measured concentrations (possibly in form of a table) can be found where? Some supplementary table?

Transcriptomics-derived constraints

1. Line 598 "extermal fluxes" → external fluxes
2. Are vL and vU in bolt?
3. The FVA is used here. Could the authors provide the Reviewer with the data of the FVA? As in how much do the exchange fluxes actually differ / how much are they constraint? Can the

authors please provide (here in the reviewer response, I don't think it is required in the article) the actual "before and after" FVA-constraints? Or provide the Code, so I can check that.

4. line 603 "only exceptions to application of this constraints" singular/plural mix-up?
5. Also these reactions: Interesting, that this is only the case for some cell lines. Could the authors please add a discussion around these points, for why this might be the case? (In the discussion on these reaction parts)

Generation of FFD dataset

1. The authors use COBRApy. Is the only setting on the optGpSampler the number of solutions? Have other functions been used, that require a citation?

Generation of RPS dataset

1. The explanation of the metabolite flux mentions in line 619 "molecules per unit of time". One of the major assumptions in genome-scale metabolic modelling is the steady state (as the authors also describe), which eliminates the concentration change in time, as there is no change in steady state. This statement should be removed.

Concordance analysis

1. "the variation in the value" → in which value? Flux? RPS?
2. "We decided to treat our measurements, i.e. the log ratio of the flux, as up, down or no-change." That means, it is similar or different from the other literature mentioned afore? And what does that mean to the log ratios of the flux? Are they only treated "binary" as -1, 0, and 1, not using the actual value?
3. Line 659 "Hence,[…]" Due to the log2?

Ranking of discriminative fluxes

1. This paragraph should be refined for better readability. (e.g. "we tried to address" → Did you or did you not address it?; derive 2x in the next sentence, which is also should be split into 2 sentences; "it is possibly to identify cell lines with a more set apart fluxes distribution with respect" → flux distribution?)

General note computational part: I think the authors should add examples for their calculations, to clarify these scores. The authors could use some reaction which may change between two cell lines and calculate most examples with these specific data as a red thread throughout the computational materials and methods sections. This would also clarify the points of "missing data/inputs" that are required for such calculations, that are obvious for the authors.

I also think, the experimental part should come first, as the models use the experimental data as input.

Experimental part

General note: There is not a single citation in the experimental section. Is the data generated somewhere accessible? At least some growth plots in the supplementary material?

Cell culture

1. What media did the authors use to maintain the cells, and what media for cell the experiments themselves?
2. What is the glucose concentration (low, high, glutamax?)

Cell proliferation

1. How many cells were plated?
2. What kind of well plates, where purchased?
3. Normal growth medium means the DMEM for the respective lines?
4. How were the proteins extracted? What are the specifics on the Bradford assay?

Metabolite extraction

1. The computational part stated the uptake rates are based on “time 0h and 48h” (line 583 for example). Here it states, that fresh medium was added after 18h…. Does that mean, that 0h is actually 18h for the computational side, and did they know/was that taken into account? I assume, that that medium was used, as nothing else is stated for the medium analysis?
2. Sidenote for future experiments: Quenching with liquid nitrogen instead of the -80 freezer?
3. 70% power for the sonicater does not provide any insight if the model is not mentioned.
4. centrifugal vacuum concentrator → speed, pressure?

LC MS metabolic profiling

1. LC gradients are strange, as they 0 min A, 2 min A, 4 min 0.99A, 10 min 0.98 A… why is the 2 min step there? Similar for 11 and 15 min, as both is 0.7 A. The 5 min post-run is with A? So total runtime per sample 21 minutes?
2. Which gases did you use?

Metabolite quantification

1. With this information I cannot reproduce the experiments. This also indicates, that only the 48h samples were analyzed and not the 0h? Where they sterile filtered? How much was used? …

RNA extraction

1. How did the authors get from “8 x 106 cells” to “each pellet”?
2. Line 738 “RNase-free” vs line 373 “RNAse-free”
3. “after purification” → How? Miniprep? Details.

RNA-Seq

1. This paragraph needs a work-through, as there are quite many “the” missing, and some sentences are long and thus hard to read.
2. Line 746 “Starting form each RNA isolated” –> isolate; “as first” → at first
3. Line 751 “diluted at” → diluted to

General material and methods:

The language and understandability is mostly good. It is possible to follow the descriptions. There are multiple citations missing and I am not able to reproduce the presented methods/work based on the description. The link (NOT provided in materials and methods) to the github (https://github.com/qLSLab/integrate) is not working (potentially a private repository?), and thus an evaluation of the proposed pipeline and the methods is also not possible. In addition the data generated in the experimental parts is missing to a large extend, and none of the data in the supplementary files is linked in the methods part.

Introduction

General note: The authors are inconsistent: In lines 42++ the examples are S1 and E1 with/without italics, with/without subscript, with/without []. Are they different? Another examples are constraint based vs constraint-based, metabolomics data vs metabolomic data.

1. "In recent years, the biological role of metabolism has been reconsidered" as first sentence is quite a statement without any literature sources. Please do add citations.
2. The pattern of missing citations for statements made continues here. I will not further highlight them, but assume, that the authors find and fix these issues themselves.
3. The authors should also consider, for better readability, to shorten sentences. For example, lines 21 to 30 (10 lines) are two sentences. There are quite a few 5-line-sentences. Also adding paragraphs would increase readability (e.g. there is none the first 35 lines). For example I had to read the intro two times to realize, that lines 30 to 35 are the foundation for the manuscript. I feel this should be more highlighted by elements such as paragraphs, but also more than a "it is worth noting".
4. The sentence "The gene expression layer sets the upper bound for any given flux" should be clarified.
5. The example of the two cells A and B and the metabolic reaction could be illustrated.
6. I suggest a change "S1 is in excess, so the flux through E1 is independent" to "the flux through r1"
7. Lines 61++ ("Yet, the integration of these […]"): There has been multiple integrations of these type of omics data, specifically transciptomics data (e.g. gxFBA; for review see e.g. https://dx.plos.org/10.1371/journal.pcbi.1003580). The authors should clarify that specifically the integration of BOTH datasets is discussed here
8. "As a proof-of-principle […]" Please also include the results of the pipeline-integration, how does it predict in comparison to the lab measurements that the authors generated?

General note: Please expand the literature review on transcriptomics and metabolomics integration into genome-scale metabolic models, specifically the well-established methods/approaches. Compare these to the proposed parts here. The whole experimental part is mentioned in half a sentence, the authors

should expand on that and potentially also link that more to the start of the introduction/give potential use cases this pipeline could impact science and society.

Results

The INTEGRATE pipeline

1. Lines 108++: I think, the authors should add/clarify, what the omics data is: Is it a single measurement of a single cell line (I like the definition in the start of the Results part), or is it measurements from two starts and taking the "delta" of these? From the output of INTEGRATE I'd assume, it's the latter, but I feel this should be clarified.
2. I was actually looking in the Methods for "the method", but since the Pipeline is a result as well, I guess, it is fine to have a detailed description here, discussing the different steps in detail. Unfortunately, the description is only scratching on the surface of the pipelines capabilities, for a suggestion see point 6 here.
3. Reactions missing a metabolite measured my metabolomics are omitted. What is about the Transcriptomics then? They are omitted as well?
4. Figure 1) RNA-seq read counts = transcriptomics?! Indicate which of the input datasets is optional. Maybe differentiate "Input, Pipeline, and Output" a little better? The model is also an input, right? And the derived Datasets are an output? I assume, since I cannot test the pipeline code, that the derived datasets are saved output, and the variation concordance analysis is an additional output? The figure description could be expanded a little.
5. Table 1) Caption below the table? Maybe include ER and PR abbreviations?
6. Line 134: "Once the three reaction-oriented datasets are obtained,[…]" I am still not sure, how that works exactly. Some short example toy-code could be nice to highlight some of the core features of the proposed pipeline.
7. Line 137 "a proper statistical test" The authors should either mention the test here, or link to a later description in the manuscript, potentially give examples they used to clarify what a "proper" statistical test is.
8. Note that the description of the proposed pipeline, which is the main result of the manuscript (as the cell tests are a test case), is merely one page of text. I feel the functionality of the pipeline and more detailed functionality deserves some more focus.

Selected breast cancer cell lines […]

1. Fig 2) Lines around the plot. Figure quality (especially D), Red and green in a figure, Alignment (A, C, and D are shifted.
2. Here is a link to "all the experimental data". Is it possible to add units? And a description of what is what? I am missing the (growth) data, the Figure 2 is made from.

The ENGRO2 model

1. The model is based on RECON3D, as the authors state, basically a curated core model of the RECON3D. Can the authors include a table with the comparing key parameters of the 3 models (recon, Engro1/2) such as number of reactions, genes,… and potentially also specifically the number of reactions within their core model, that they added/curated in comparison to the recon3/Enrgo1/HMR?
2. I would have liked to see the explanation of lines 211-216 in materials and methods, as this actually explains the authors general model reconstruction steps. I suggest moving these lines, as they are actually materials and methods (or a discussion of the model/results for that matter) but not results. That said, I think almost the whole chapter should be Discussion as the authors thoroughly discuss the model generation and the reasoning for it. However, the authors do not present the model per sé, as for example I suggested in the previous comment: How many reactions does the model have? Genes, GPRs, and other model typical parameters.
3. Side note: "Recon3D" or RECON3D?... PLEASE be consistent!

Cell relative metabolic models

1. I do not agree with the "Constraints on nutrient availability" as more available nutrient will not necessarily increase the uptake. For example, an Ecoli cannot take up more than 16mmol/gCDW glucose even if the amount of glucose would be increased 10-fold. For lower concentrations, up to the limit this is reasonable, however. Hence, I suggest that the authors include a cap for what is biologically possible/plausible, and potentially lower the upper bond of the other cell instead?
2. Lines 279-283 are discussion, no results.
3. Line 284: "In Fig. 3 it is evident […]" The authors should be careful with these statements, as it might not be evident for every reader. Further, the "iterative application of the 3 constraints" -> Which of the methods did the authors apply iteratively, and how many times? That as well as the implementation should also be mentioned in materials and methods.
4. Fig 3: Red/green; "evaluation of the iterative […]" suggestion to include "how" – e.g. mention tSNE; The figure is 2 Panels with 4 subfigures each. The authors list "type 1" in the description, and have a black box with a "1" in the figure. Suggestion to make that A1 A2, A3 maybe? Or add Boxes around A and B and use a headline for the figure, e.g. "nutrient change", "extracellular fluxes",.. to make it easier to understand; Why did the authors not include the metabolomics?; wet -> in vivo; What is growth yield?
5. Line 290 "decrease the distance" -> What kind of distance measure was used? I assume, that the authors mean a clearer separation in the plots, and they should maybe reformulate that.
6. Line 292: "growth yield" What do the authors mean? Growth rate? Number of cells? Cell Dry Weight production? How did the authors measure/calculate these (Move lines 293-297 to materials and methods). Can the authors potentially also have these values in a table?
7. Line 293 "wet and computational data" Please reformulate that…. I know what the authors mean, but "wet data" is a formulation that makes me smile a little, which we would also (and only) say to/about "wet-lab-people".
8. So far the authors did "only" discuss transcriptomics and secretomics data with the use of the model. (I would consider the matching of experimental medium to the computational medium a normal modelling application) Why did the authors not include their metabolomics data and evaluate the full potential of INTEGRATE?

INTEGRATE discriminates reactions […]

1. Here are again parts that are materials and methods (e.g. Cohen's Kappa coefficient) as well as discussion.
2. Fig. 4: red/green (and that in combination with grey) Alignment of the plots, as well as the quality. Placement of the color bars, only description of one of them. "cohen", "ACONT" – These require a description what they are. Remove the sentence-repetition.
3. Can the authors include a table with the concordance values and the numbers of reactions that score with the respective values?

A few fluxes well […]

1. "The ACONT reaction, which we previously discussed"; The discussion continues in line 366. I suggest moving these points to the discussion and keep to the results in the results section.
2. Fig. 5 A box around the figure; Flux value [unit]; RPS/FDD black, ACONT/OCOAT1m grey; "according to the flux of cytosolic ACONT" -> reformulate; 5 -> five; "C) Same as B" I would repeat the description, but I guess due to the figure size, that is fine.
3. Line 371: suggest to remove "in Figure 5"
4. Line 373+ "similar to what was done in 32" Add a brief description into materials and methods. If it only was "similar" and not the same way, please state the differences so the work it is reproducible.
5. 389 "only in the extent" -> to the extent
6. Fig. 6: red/green; please us the real amino acid names, not the model metabolite IDs; Why not integrate the AA name into the y-Axis description? For most of them, the orange one (MCF102A) is hardly visible. Is it possible to highlight it somehow?; The figure description is vastly different from the previous ones, please be consistent. I think, it is a good idea to give a general explanation , but I think, the AAs should be mentioned earlier.
7. Side question: Did the authors change the biomass? Since the contribution of the AAs to the biomass does not change, if the authors did not change the biomass coefficient. Hence, the contribution to biomass would directly correlate to the growth of the cells. The plotted uptake of AA and directly correlates with the growth (I assume, the biomass coefficient for the specific AAs were not variable) and I assume what is visible with the 1M samples is basically the flux variation in that correlation. Please do keep during the discussion (lines 380-387) in mind, that the models assume steady-state and mass conservation.

General comment results:

The authors mix results, materials and methods, and discussion for many parts. The result part should be a listing of results, which then are discussed in the discussion, if these are different chapters. I wonder why the authors did not evaluate all parts of INTEGRATE, as they do not appear to evaluate the impact of the generated metabolomics data and thus loose the advantage over other extended transcriptomics integrations. The authors also do not show a direct evaluation of the pipeline, e.g. is it

better then other pipelines/easy to use methods, what is their advantage, how does it correlate to experiments?

Discussion

Lines 392-421 feel like a repetition of the previous results parts, whereas 422-427, 446-449,464-470 feel like a conclusion.
