# Supplementary material for: INTEGRATE: Model-based multi-omics data integration to characterize multi-level metabolic regulation
Source: PLoS Comput Biol. 2022 Feb 7;18(2):e1009337. doi: 10.1371/journal.pcbi.1009337 (PMC8853556; doi:10.1371/journal.pcbi.1009337)
Supplement: Attachment — Submitted filename: Reply.pdf [file pcbi.1009337.s013.pdf]

Milan, December 4th 2021

Dear Editors and Reviewers,

please find enclosed the revised version of our manuscript entitled "I*NTEGRATE: Model-based multi-omics data integration to characterize multi-level metabolic regulation*", submitted to **PloS Computational Biology**.

We would like to sincerely thank the reviewers for the in-depth analysis of our work and for the useful comments.

We made an extensive effort to address all the concerns and comments raised by the reviewers, and this led to a substantial rewriting of the manuscript. In particular:

- we re-executed all simulations to guarantee more biologically grounded growth yields
- we included a new section to better assess the robustness of our results
- we showed a new proof of principle of our pipeline by using the genome-wide model Recon3D
- we substantially rewrote the Material and Methods section to make it more independent from the Results section, and to make results fully reproducible
- we better clarified the novelty of our work with respect to the state of the art
- we included toy examples to better explain the steps of the pipeline that we are proposing

For your convenience, any change and new text is marked in red, but we also provided an unmarked version of the manuscript.

A point by point response follows in the next pages.

We believe that these extensive revisions have largely improved the overall quality of the manuscript.

Kind regards,
Chiara Damiani,
also on behalf of the other authors.

Reviewer 1

Review summary

The submitted manuscript by Di Filippo et al. integrates transcriptome and metabolome data with stoichiometric models to unravel whether specific metabolic fluxes are governed by transcriptional regulation, differences in substrate levels, or both. The authors apply their method to study a set of cancer cell lines that have a clear difference in growth rates, substrate uptake and secretion of glutamate and lactate, intracellular metabolite concentrations and gene expression levels. Their main finding is a description of how the two different modes of flux control affects a set of specific reactions in the core metabolism of cancer cells. The study includes further development of a core metabolic model for cancer cell lines with about 500 reactions and metabolites.

The proposed method (INTEGRATE) uses relative gene expression levels (translated to "reaction activity scores (RAS)" through GPR associations) to scale metabolic fluxes in each cancer cell line relative to the maximum flux identified using flux variability analysis (linearly). Then, they apply constraints on exchange fluxes according to measure substrate consumption and secretion of lactate and glutamate (as relative yields), and other differences metabolite availability as dictated by the different cultivation media used for the different cell lines. The authors then sample the solution spaces for the different models (corresponding to the different cell lines) to obtain "feasible flux distributions" (FFD). In parallel, the authors compute "reaction propensity scores (RPS)" for each reaction in each model (the product of substrate concentrations). The authors then perform pairwise comparisons of FFD, RAS and RPS scores for each reaction to investigate whether these values are significantly different between each pair. These scores are then used to calculate the agreement (or concordance as it is called in the manuscript) between FFD and RPS, and between RAS and RPS.

The underlying hypothesis is that the agreement (concordance) of FFD-RPS and RAS-RPS can be used to distinguish whether a reaction is controlled by transcriptional regulation or by substrate levels.

I find the hypothesis interesting and valuable and as such, I think the manuscript provides value to the COBRA community.

However, my main concern is that the manuscript does not validate the method, and it is therefore yet to be seen whether the main hypothesis is true and if the proposed method can provide valuable predictions. Although lack of proper experimental validation is (unfortunately) not uncommon in this field of research, I'm particularly concerned about experimental validation when transcriptome data is used to infer reaction fluxes (see review by Machado and Herrgård, PLOS Comp. Biol, 2014). Furthermore, and as the authors point out themselves, the underlying assumptions (linear relation between substrate concentrations and flux and linear relation between transcription (RAS) and flux) are not always valid, and hence, it suggests that some validation is needed to determine the usefulness of the proposed method.

We thank the reviewer for the constructive criticism. We appreciated that he/she fully captured the overall workflow and idea.
We understand the reviewer's concern about the lack of validation, in general and specifically for our methodology. Validation of our results is indeed not straightforward, as it requires ad hoc experiments and dedicated funding.
We are also aware of the review by Machado and Herrgård and we share the reviewer's concern about using transcriptome data to infer reaction fluxes. For this reason, we took some precautions that should mitigate prediction errors. First of all our approach does not have the ambition to predict exact flux values, but rather the sign of the variation across the cell lines. Moreover, we are using transcriptomics data on top of constraints on extracellular fluxes and on growth. Constraints on extra-cellular fluxes and metabolic growth, along with the mass balance constraint, relax the assumption on the linear relationship between gene expression and flux capacity constraints. We are afraid we did not make clear that our approach does not strictly assume a linear relation between substrate concentrations and flux. On the contrary, we classify as metabolically regulated reactions, without the ambition to be exhaustive, reactions in which a monotonic relationship between substrate and flux takes place. Along similar lines, we classify as transcriptionally regulated reactions, again without the ambition to be exhaustive, reactions in which a monotonic relationship between substrate and flux takes place.
Unfortunately constraint-based models are (by definition) sensitive to constraints, so the affiliation of a reaction to a given class may be affected by the choice on how the constraints are imposed and on the model stoichiometry, as well as by the way in which the feasible region is explored. In the revised manuscript, according to reviewer's advice and as detailed in the following, we performed new statistical analyses to better assess the robustness of our results.
All in all, in spite of its limitations, as the reviewer recognizes, we think that our proposal can be a good fuel for thoughts in the Cobra and cancer metabolism community.

We discussed the above limitations of the approach and clarified our assumptions in the revised manuscript (section 'Discussion and Conclusion'.).

Major concerns

- The github repository (https://github.com/qLSLab/integrate) is not publicly available, and it is therefore impossible to evaluate the associated code

We truly apologize for this oversight. We have now made the github repository public.

- The authors use a designated core model. It would be very valuable to understand how sensitive the proposed method is to the model structure. This could potentially be tested by either making a set of core models integrating different aspects of a full recon3D / HMR model, or by applying their method to Recon3D, HMR, or HMR core model (possibly contextualized using the transcriptome-data) and observe if the method infers similar regulatory mechanisms. Similarly, application of the method to an additional species/context could display generic applicability of the method.

To roughly assess the sensitivity of INTEGRATE results to the input metabolic model, we re-executed the overall pipeline on RECON3D, by limiting the model to the set of exchange reactions that is active in ENGRO2. Results are reported in Figure 5 of revised manuscript. First of all, it can be observed that, also in RECON3D reactions distribute along the three categories.
Moreover, the comparison of the distribution of the concordance scores (Figure 6) against the random case resembles the ENGRO2 case (Figure 4C) confirming that in both models a few fluxes agree with RPSs beyond reasonable doubt.

As expected the sets of reactions identified by the two models do not coincide. Yet some overlaps can be observed. For example the RPI reaction appears to be “transcriptionally and metabolically regulated” in both models.

These results are discussed in the new Section titled “Robustness of INTEGRATE results”.

- According to the diagram in Fig. 1 there are three potential regulatory options: metabolic, transcriptional and both. However, as the method does not account for allosteric regulation, product inhibition, or cofactors / prosthetic groups, one would anticipate that one could not pinpoint the regulatory mechanism for all reactions, i.e. should it be an "other" category in this diagram? Furthermore, the upper left quadrant is not explicitly mentioned, but there are reactions in this quadrant in Fig 4A. Can the authors elaborate on how one should interpret these results?

We thank the reviewer for this comment. Indeed, we observed some reactions having positive values for RPSvsRAS but negative RPSvsFFD scores. The phenomenon according to which gene expression and substrate availability agree with one another but the flux does not agree with any of the two might be imputed, as the reviewer suggests, to allosteric regulation, product inhibition, or cofactors/prosthetic groups. Yet, it might also be due to inaccuracies in model predictions. In order to exclude the second hypothesis, other kinds of omics data would be necessary. Yet, in our case study, only a few reactions belong to this category and with statistically not significant concordance levels. Hence, we followed the reviewer’s advice and simply labeled the second quadrant as 'other'. We discussed the meaning of this quadrant in the revised manuscript.

- Although the methods used to generate results in Fig. 5 and 6 are elegant, I think the last part of the results and discussion could be shortened. I think it would be better to clearly summarize / show / verify the proposed method rather than exploring other metabolic differences between the cancer cell lines in such detail. Maybe Fig. 5 and 6. Could be combined into one, as a short proof-of-concept figure summarizing the metabolic differences between the cell lines?

We agree with the reviewer that these analyses, even though interesting, are a bit off topic. We decided to completely remove this part about downstream analyses from the manuscript and just mention them in the discussion section. In its place we inserted a new section and Figure that assess the robustness of INTEGRATE results.

- On page 17, line 425 the authors write "Remarkably, we identified reactions for which there is a good agreement between flux variations and variation in RPSs". If the metabolomic data or transcriptome data was completely random, how many reactions with an agreement between RPSs and flux variations is expected just by chance?

We thank the reviewer for this comment. Although Cohen's kappa takes into account the possibility of the agreement occurring by chance, when assessing Cohen's kappa of multiple reactions, the probability to observe a high Cohen's Kappa value by chance is expected to increase. Traditional methods for multiple comparisons adjustments focus on correcting the p-values of the statistical tests. Given that Cohen's Kappa does not return a p-value, in the revised manuscript, we associated an empirical p-value to the obtained Cohen's kappa v values.

To this aim, we obtained the empirical probability of agreement between two independent datasets as follows. We randomly sampled (with replacement) the distribution of RPS variation values 100 times. We sampled directly the results of the statistical tests (-1,0,+1), otherwise when performing the tests with random data we would have had maily 0 values.

The comparison in Figure 4C (revised manuscript) between the distribution of the concordance values obtained with INTEGRATE and the empirical probability of agreement between two independent datasets confirms that a few fluxes agree (or disagree) with RPS variations beyond reasonable doubt. On the contrary, for the majority of flux variations the agreement is not significant. The non significance can indicate that FFDs and RPSs do not agree, as well as that the agreement is uncertain. We believe that his uncertainty mainly depends on the limited number of cell lines for which we are assessing the agreement. Other possible sources of uncertainty are the uncertainty in the experimental data, the strictness of the mass balance constraint and the limited scope of the ENGRO2 metabolic network.

To deal with the multiple comparisons problem, we then associated an empirical p-value to our obtained Cohen's kappa values and adjusted it with the Benjamini and Hochberg procedure to keep the False Discovery Rate (FDR) below 5%.

After FDR correction of the p-values, the concordance between RPSs and FFDs resulted statistically significant for four reactions. It is however important to mention that FDR correction is a standard practice in gene expression analyses, but it might be too stringent in

our case. In particular, one should take into account the lower probability of simultaneously observing a high RPS-FFD concordance and a high RPS-RAS concordance by chance.

It is also important to mention that FDR correction is a standard practice in gene expression analyses, but it might be too stringent in our case. In particular, one should take into account the lower probability of simultaneously observing a high RPS-FFD concordance and a high RPS-RAS score by chance.

We discussed the above results in a new section of the paper titled "Robustness of INTEGRATE results".

- How do you ensure that the flux sampling don't provide unrealistic flux values caused by loopsn you split all reversible reactions into two irreversible?

In order to set meaningful transcriptomics-derived constraints, which in turn depend on FVA results, we performed FVA before rather than after splitting the model. By doing so, the upper bound of the backward and forward reactions in the splitted model will be at most up to the respective net flux. Let us clarify this with an example: if in the original model the maximum flux of the forward reaction is 100 and that of the backward is 0, in the splitted model they might be 1000 and 900 respectively. Yet we will constrain the forward reaction to be at most 100 (scaled on RAS value) and the backward reaction to be 0. This does not guarantee that the sampled flux values are completely realistic, but we are confident that statistically significant qualitative flux variations (up, dow, or no change) are realistic.

We stressed in the revised Methods section that the model splitting must be performed necessarily after and not before defining type 2 constraints, to avoid basing them on unrealistically high FVA values caused by futile loops.

Please also notice that in downstream analysis, when possible (as e.g. for clustering plots), we consider the net flux (forward minus backward).

- Figure 3B: It seems to be a difference between the computational and experimental yields on the scale of two orders of magnitude. Why?

In view of our "relative approach", according to which all constraints on fluxes were set using relative values rather than exact values, at first instance, we did not pay particular attention to exact yield values, but we merely focused on the correlation between experimental yields and computational ones. Yet, we agree with the reviewer that a two order of magnitude difference is quite peculiar and hence we investigated the issue.
First of all, we paid more attention to make the two yield values (exp and comp) truly comparable. In fact, in the original manuscript, we were considering the biomass flux over glucose flux and correlating it with protein flux over glucose flux. We now consider only the biomass fraction corresponding with aminoacids. Moreover, we were not converting glucose into grams in the computational case.
Yet, fixing the above problems did not completely solve the order of magnitude problem and we could not identify the cause. For this reason and because of the reviewer's minor concern about using 1e-4 as a lower bound for growth, we decided to constrain growth

yields to be closer to experimental ones and remake all the simulations. Nevertheless, we did not want to impose a specific growth yield to each cell line, because we wanted to investigate whether differences in growth yields emerged from the other relative constraints. Hence, we now constrain the growth yield of all five models to be within the minimum and maximum growth yield experimentally observed across all samples.

Specifically, we set the yield constraints as described in the revised Methods section (when describing FVA in the “transcriptomics-derived constraints” section), and as reported in equation 6.

We also specified in the flux sampling section that we retain the constraints on growth yield during the flux sampling.

We remark that, given that the correlation between experimental and growth yield is not extremely high, when applying the pipeline one may want to constrain cell-specific growth yields. Given that constraining the yield did not overturn our results, we do not expect our results to be particularly affected by such change either.

Minor concerns and comments:

- Abstract: It seems more logical to present transcriptional regulation as the first class of regulation and regulation as the second class, and I think it will be easier to read if presented in this order.

We rearranged the paragraph according to reviewer's suggestion

- Figure 2D. Color scale could be diverging from 0 to better display above and below average. The meaning of the colorbar-legend is also somewhat unclear, as abundances can't be negative. Is it Z-scores?

We thank the reviewer for the comment. It was actually a z-score. For better readability, we have now normalized each row of the dotplot by the maximum average of all the cell lines, instead of using the z-score. We clarified this point in the Figure caption. We also followed the reviewer's advice and changed the colormap.

- Figure 2F. I think it would be easier to interpret a normal semilog (log10) Y-axis scale. Also, error bars are difficult to see.

We improved the plot according to reviwer’s suggestions.

- Discussion: Can you discuss whether the current framework can be extended to also cover allosteric and cofactor regulation?
- Discussion: Can proteomics be used rather than transcriptomics to predict RAS scores? Would the authors anticipate better results from proteomics rather than transcriptomics?

We have discussed both points above in the revised conclusion section as follows:

The direct exploitation of metabolomics data to determine whether a flux is regulated at the metabolic level represents the main novelty of our approach. At first instance, we intercepted this information with information on transcriptional regulation. Nevertheless, the pipeline can be promptly extended to integrate proteomics and phosphoproteomics. One might indeed compute (phospho)proteomics-derived RASs, along with transcriptomics-derived RAS. Proteomics-derived RASs are expected to reflect enzymatic activity better than transcriptomics-derived ones, yet the latter offer better coverage. For this reason, one should still use transcriptomics-derived RAS to constrain the metabolic fluxes of reactions for which quantification of any protein involved in its GPR is not available. One can then evaluate a posteriori the concordance of fluxes with either proteomics-derived or transcriptomics-derived RAS, to identify more hierarchical regulatory layers, widening our understanding of metabolism. We remark that extension of the pipeline to cover allosteric and cofactor regulation would require explicit modeling of kinetic dynamics, which in turn requires knowledge of kinetic parameters. Alternatively, one might indirectly identify them by exclusion, that is, by supposing that fluxes that do not appear to be regulated by none of the other regulation levels are controlled by allosteric effects, or by looking for correlations between fluxes and metabolites within the same pathway or belonging to other cross-related biochemical pathways.

- Author summary: proteomics can also give clues on metabolism, not only metabolomics and transcriptomics

We agree with the reviewer. However as of today, transcriptomics is much more used than proteomics. As discussed in the previous point, proteomics could substitute or complement transcriptomics. We rephrased the sentence in the Author summary as follows:

"Metabolism can be studied using post-genomic technologies. Transcriptomics, proteomics and metabolomics provide snapshots of transcripts, enzyme levels and metabolites in specific physio-pathological conditions. In the health field, the transcriptome and, more recently, the metabolome have been broadly profiled at the pre-clinical and clinical levels, while only more recently proteomic studies profiling metabolic enzymes are becoming available."

- Line 42: typo, transcriptional control

We corrected the typo.

- Line 42-50: Rewrite for clarity. I suggest considering the flux through the reaction r1 rather than enzyme E1. Last bullet point should say something along the lines: "flux through r1 is co-regulated with changes in [s1] and [E1].

We substantially rewritten this paragraph.

- Line 67: Suggest citing some of the seminal papers or reviews on integration of GEMs and omics data her.

We included some references.

- Line 71: Unsteady FBA should also be cited here.

Thanks for the suggestion. We included the reference.

- FVA: Using 1e-4 as a lower bound for growth will result in somewhat unrealistic flux boundaries. If you use a value closer to the measured growth rates I expect that you would get more realistic flux bounds. How does this affect downstream analysis?

As discussed above we now constrain the growth yield of all five models to be within the minimum and maximum growth yield experimentally observed across all samples.

- Line 107-108: combine into one paragraph.

Thanks for making us notice this.

- For how many reactions in the models were you able to calculate a RPS?

We have 81 reactions out of 533 reactions for the irreversible model, for which we are able to compute the RPS. Note that we computed the RPS for those reactions for which we have the information about all the substracts of the reaction.
This information was redundantly present in the original manuscript.

- Line 145: Typo and rewrite: E.g. Reactions … high FFD-RPS are under metabolic control.

We corrected the typo.

- Figure 4A: Should the figure display a boundary at 0.2 concordance, as this is considered poor concordance? Also, write out proper x- and y-axis labels.

Thank you for the suggestion. We have inserted rectangles in the figure to display the boundary at 0.2 and -0.2 concordance for axis x and y, respectively.

- Figure 4B: Maybe have a separate column with reaction name / id to make it easier to read.

Thank you for the suggestion. We have splitted the column into a reaction name column and a formula column.

- Figure 4C: Not really useful.

We removed the figure.

- Figure 5B/C: Mention that color of names corresponds to flux distributions to the right.

Thanks for the suggestion. We mentioned this in the caption of the Figure.

- Figure 6: Although exchange fluxes are indeed negative in GEMs when substrates are consumed, it might be easier to interpret this result if positive values are used, labelled substrate uptake. Furthermore, the y-axis label seems strange; assuming that the biomass coefficients is fixed it is a constant relative abundance of each specific amino acid. Isn't the y-axis here basically the flux drain to growth, also in mmol/gDW/h?

As mentioned above we removed those plots.

- Figure 5B/C: Fluxes seems unrealistically high

As compared to maximum uptake of carbon sources (e.g. 25 for glucose) they do not seem that high to us.

Reviewer 2

First of all, we would like to sincerely thank the reviewer for the in-depth analysis of our manuscript. We made an extensive effort to address all his/her concerns. We provide a reply to each comment below.

Here summarize below the main improvements:

- w extensively modified the Material and Methods section to make it more stand-alone and to make the experiments fully reproducible.
- We made all scripts and (raw) data available. We truly apologize for the github being not public, this was an oversight
- We extended the Introduction section to better explain the novelty of our work with respect to the state of the art and to better motivate why we do not use metabolomics data as constraints in constraint-based modelling. We also clarified that our aim is not to present a new method for the integration of transcriptomics data into constraint-based modeling. On the contrary, the pipeline we are proposing is mainly independent from the choice of the algorithm used to customize the model according to transcriptomics data and extracellular fluxes. Our novel hypothesis is that the concordance between fluxes and RPSs (that we compute from metabolomics data in a novel way) can be used to distinguish whether a reaction is controlled by transcriptional regulation, by substrate levels, or both.
- We added toy examples and diagrams to better illustrate the pipeline steps
- We streamlined the writing of the Introduction section
- We improved the separation between Material and Methods and Results sections

Materials and Methods

I like to start reading this part of a manuscript to evaluate, whether I can understand the experiment and line of though based on the abstract. Further, I want to evaluate, whether I could reproduce the experiments based on the descriptions.

General note: The authors are inconsistent: For example, in line 524 the "S matrix", and in 528 "matrix S". In the experimental part, where µL is in italics, whereas all other units are not (nM, mL). The authors should also proof-read the whole section, as e.g. line 736 mentions "at least 8 x 106 cells" and line 696 "CO2", which are easy errors to spot.

All the authors carefully proofread the revised article and removed inconsistencies.

Model reconstruction:

1. Which program was used, which version, functions to be cited,…?
We clarified that the overall reconstruction process was entirely manual

2. Which model was actually used? ENGRO2 or 1, or construct a new one based on both? Are these published models? (as there is no citations) In the supplementary is the ENGRO2 model listed, does that include the changes/integrations made in this manuscript? Can the authors please clarify which model the authors are discussing about, e.g. name the model used in the manuscript ENGRO2_met_tra for easier distinction.

We have now specified all these details also in the Methods section.

3. Based on the text in the chapter "Model reconstruction", I would not be able to reconstruct the model. E.g. "it was necessary to add the ROS detoxification pathway by means of glutathione" or "we enriched the ENGRO2 model by including both folate and the methionine cycles" does not tell me anything about actual changes to the model.

We have now included a human-readable version of the model (besides the SBML) in Excel format, so that the formula of reactions included in each pathway can be readily identified. See supplementary file S3.

4. How does the Biomass reaction look like based on the last-paragraph description? I assume, the authors removed the mentioned compounds, added the molar mass equivalent to the biomass and rescaled the whole biomass function?

We have better clarified that the biomass synthesis reaction was exactly copied from Recon3D model in terms of metabolites and stoichiometric coefficients. yet, given that ENGRO2 does not explicitly include lipid synthesis, we assigned the sum of the stoichiometric coefficients of 1-Phosphatidyl-1D-Myo-Inositol, Phosphatidylcholine, Phosphatidylethanolamine, Phosphatidylglycerol, Cardiolipin, Phosphatidylserine, Sphingomyelin to palmitate.

FVA:

1. FVA was not developed by Gudmundsson and Thiele.

We thank the reviewer for raising this comment. We have now properly referenced FVA [Mahadevan, R., & Schilling, C. H. (2003). The effects of alternate optimal solutions in constraint-based genome-scale metabolic models. Metabolic engineering, 5(4), 264-276.]

2. The one sentence summary is hard to understand, something along the lines of “FVA determines the maximal (and minimal) flux possible through any reaction of the model and thus evaluates the cell’s range of metabolic capabilities.”

We extended the discussion on the rationale for using FVA in our approach and moved the overall FVA description to the Section named ‘Trascriptomics-derived constraints - type 3’.

3. The statement “As other constraint based modelling methods, it relies on the fact that the steady states of the metabolic model corresponds to the kernel of the stoichiometric matrix S associated to the model itself” should be supported by citations. The next sentence reads “It is exactly this null space that defines the space of all the feasible fluxes.”, which makes me wonder about the null space, as this is not mentioned before. (This is complaining on a high level though, I apologize for that)

We are afraid we gave too many linear algebra concepts for granted in the original version of the paper. In the revised manuscript, we substantially rewritten all the parts concerning FBA/FVA. In particular, we inserted a lead paragraph at the beginning of the section “From ENGRO2 to cell-relative constraint-based models” to better explain these concepts.

4. I do not understand the following sentence: “Moreover, to mimic as closely as possible the biological process in analysis, it is possible to bound the feasible fluxes space by means of convex half planes represented by the reaction's lower and upper bounds.” I think, here is some English-language fixing necessary, and a citation.

We rephrased the sentence and referenced related work.

5. Since this is “materials and methods”: The method was FVA, but what did the authors do with the FVA? And how? In which program?

As mentioned above, we removed this section, as it was indeed misleading, and introduced FVA in the section on Trascriptomics-derived constraints, where we actually use it.

6. I am not sure, whether the usage of FVA is correctly in this manuscript. Usually (as the authors state) one would use FVA to determine the variable fluxes given a percentage flux trough the objective function. Relaxing that percentage will result in broader variable flux ranges, as all reaction fluxes can be redistributed fulfilling the objective flux requirement. The authors state here that they only want to prevent a non-zero objective function flux. This should relax all other fluxes to their absolute maximum, so basically, determine what the network restrictions are in a given environment. Would this actually constrain the model

fluxes (sure, it might constrain the lb/ub of a given reaction instead of -1000/1000, but they would be constraint by the medium to the same values, right?) glielo spiega meglio CHIARA

The reviewer correctly interpreted that we used FVA to determine what the network restrictions are in a given environment. We better discuss this point by adding the following paragraph:

“In order to properly limit a reaction flux based on its RAS relative score, as a preliminary step, the maximum flux through such reaction, based on extracellular flux constraints (i.e., type 1 and type 2 constraints), must be determined. In fact, limiting the simply theoretical upper bound (i.e. 10.000) might have no effect if the actual flux capacity is lower than the former.”

Yet, if we correctly understood what the reviewer is asking, the determined flux capacity won’t be the same for all model reactions. Let us make just a rough example: if glucose uptake is 1, then the maximum flux through glycolysis will be 2, whereas the flux through respiratory chain can be up to ~32 (or more if other carbon sources can be oxidized).

Reaction activity Score computation

1. GPR rule description (line 542++) Please add a citation for the mentioned GPR rules From microbe models, the GPR rules are for genes, where multiple (linked by these rules) can encode for one reaction (protein). AND means then that both genes are needed (in case of knock out relevant), OR means that either is needed. Please include a citation for GPR rules on an enzyme level, also encoding for multimeters.

We have now referenced the use of GPRs in literature.

2. “According to [31]” Please incorporate a one to two line summary of the method. Please also formulate how the RAS is (mathematically as equation) defined.

We thank the reviewer for the suggestion. We have added the intuition and formulas about RAS computation (Eq. 1 and 2) in the new version of the manuscript.

3. Line 560 “in all the cell line” add the plural “cell lines” OK
We corrected the typo.

4. “consider that the corresponding
We reformulated the sentence.

Constraints on nutrient availability
1. The description of uptake- and secretion fluxes is great. However, this section (in materials and methods) does not provide the reader with any information about the medium used for the models, (reactions), nor any of the uptake fluxes.

We have now referred the reader to the supplementary material for extracellular flux ratios. The medium is described in the experimental section. For further details the 5 cell relative models in supplementary material include all the constraints.

Constraints on extracellular fluxes

2. Reading on, I understand that the previous section is an introduction for this one, with a different headline?

Although both types of constraints deal with the boundaries of exchange fluxes they deeply differ. Type 1 just defines the growth medium of the cell, by setting the upper bound of nutrient uptake. Type 2, set the ratio between consumption and production of nutrients. The two types of constraints coexist in parallel. We stressed these differences in the revised version of the manuscript.

3. The authors use the ratios of three pairs of metabolites to determine exchange rates. What were all the other exchange rates? Zero?

As clarified above, these constraints are additional to type 1. If no type 2 constraint is set, type 1 still holds.

4. To clarify: The authors use two pints (zero and 48 hours) to determine uptake rates (which is way too little data points, hence the authors use the ratios?). The authors did measure two concentrations (for a given metabolite at both time points), and calculated the rate… how? This needs information about the number of cells and cell dry weight. I assume, I'll read about these measurements later, however a link to the later methods part would be nice.

We thank the reviewer for raising this comment. As we mentioned in the original manuscript, there is no need to divide by the number of cells, as the ratio will always won't be affected. We have better stressed this point in the revised manuscript, remarking that we are not computing rates, we are computing ratios.
Regarding the number of time points, we remark that we are assuming a steady state in this time window, as motivated in the results section. The choice of using ratios is motivated in the results and has to do with the limited set of measured exo-metabolites.

To address reviewer concerns, we also moved the experimental methods ahead of the computational ones.

5. Also: These calculated data as well as the measured concentrations (possibly in form of a table) can be found where? Some supplementary table?
All experimental data are in Supplementary File 1 (Excel table). We have better referred the reader to this file throughout the revised text.

Transcriptomics-derived constraints

1. Line 598 "extermal fluxes" external fluxes
When still applicable, all reported minors have been corrected.

2. Are vL and vU in bolt?

Yes vector names are in bolt, following standard vector notation

3. The FVA is used here. Could the authors provide the Reviewer with the data of the FVA? As in how much do the exchange fluxes actually differ / how much are they constraint? Can the authors please provide (here in the reviewer response, I don't think it is required in the article) the actual "before and after" FVA-constraints? Or provide the Code, so I can check that.

We included in the github (folder "outputs") a file reporting the FVA of each flux. Before FVA-constraints the flux boundaries are simply -10000 for lower bounds different from 0 and +10000 for upper bounds.

4. line 603 "only exceptions to application of this constraints" singular/plural mix-up?

We corrected the typo.

5. Also these reactions: Interesting, that this is only the case for some cell lines. Could the authors please add a discussion around

We specified in the revised manuscript that the RAS score of CARPEPT1tc reaction is null in MDAMB231 and MCF7 cell lines, whereas the RAS score of HIStiDF reaction is null in MCF7 cell line. This observation derives directly from gene expression data. The reactions are essential (in ENGRO2 model) for all cell lines.

Generation of FFD dataset

1. The authors use COBRApy. Is the only setting on the optGpSampler the number of solutions? Have other functions been used, that require a citation?

We thank the reviewer for the comment. We have now specified that we used optGpSampler with default parameters, except for the thinning value that we set to 10. Because we used mainly standard python libraries, we referenced functions only when necessary, that is, when the choice of a different function in the implementation of the pipeline may potentially affect results.

Generation of RPS dataset

1. The explanation of the metabolite flux mentions in line 619 "molecules per unit of time". One of the major assumptions in genome-scale metabolic modelling is the steady state (as the authors also describe), which eliminates the concentration change in time, as there is no change in steady state. This statement should be removed.

The time change of metabolites concentrations is indeed zero, implying that the flux value does not change in time. Yet the flux value is still expressed over time. A constant glucose influx of 5mM per hour is different from a constant glucose influx of 5mM per day.

Concordance analysis

1. “the variation in the value” in which value? Flux? RPS?

We thank the reviewer for the comment. We reformulated the sentence.

2. “We decided to treat our measurements, i.e. the log ratio of the flux, as up, down or no-change.” That means, it is similar or different from the other literature mentioned afore? And what does that mean to the log ratios of the flux? Are they only treated “binary” as -1, 0, and 1, not using the actual value?

The writing of this section was indeed confusing and the literature was not referenced at the right place. We rewrote this section to clarify that we treat variations as categorical (-1,0,1). Then we use a metric standardly used in literature for the concordance of nominal variables.

The rationale for using qualitative variations is to avoid assuming proportionalities between RPS and FFD, nor between RAS and RPS values, as motivated in the Results section.

3. Line 659 “Hence,[…]” Due to the log2?

we replaced “Hence” with “Finally”

Ranking of discriminative fluxes

1. This paragraph should be refined for better readability. (e.g. “we tried to address” Did you or did you not address it?; derive 2x in the next sentence, which is also should be split into 2 sentences; “it is possibly to identify cell lines with a more set apart fluxes distribution with respect” flux distribution?)

We thank the reviewer for these comments. We streamlined the writing of this section, but then we decided to remove this part from the paper, as suggested by reviewer 1.

General note computational part:

I think the authors should add examples for their calculations, to clarify these scores. The authors could use some reaction which may change between two cell lines and calculate most examples with these specific data as a red thread throughout the computational materials and methods sections. This would also clarify the points of “missing data/inputs” that are required for such calculations, that are obvious for the authors.

We thank the reviewer for the suggestion. We included a toy model example illustrating all the steps to obtain the concordance scores, including RAS and RPS computation in Supplementary Figure S4.

I also think the experimental part should come first, as the models use the experimental data as input.

We followed the reviewer’s suggestion and moved the part related to the experiment methods ahead of the computational methods.

Experimental part

General note: There is not a single citation in the experimental section. Is the data generated somewhere accessible? At least some growth plots in the supplementary material?

As mentioned above all generated data are provided in the Supplementary Material. Growth plots can be found in the Results Section.

Cell culture

1. What media did the authors use to maintain the cells, and what media for cell the experiments themselves?

The media used to maintain the cells and the media used for experiments are the same and we have now clarified in Materials And Methods section Cell culture.

2. What is the glucose concentration (low, high, glutamax?)

The glucose concentration is 25 mM in DMEM medium and 17.5 mM in DMEM/F-12 medium. We included this information in the revised manuscript.

Cell proliferation

1. How many cells were plated?
2. What kind of well plates, where purchased?
3. Normal growth medium means the DMEM for the respective lines?
4. How were the proteins extracted? What are the specifics on the Bradford assay?

We have now provided all required information in the revised version of the Cell proliferation section in Materials and Methods that we report below.
"Cells were plated in 6-well cell culture plates (cat no. 657 160, Greiner Bio-One) in their respective growth medium previously described. In particular, 80.000 cells/well were seeded for MCF7 and MDA-MB231 cell lines, 120.000 cells/well for SKBR3 and MCF102A, 220.000 cells/well for MDA-MB361. Culture medium was replaced after 18 h and cells were collected and counted after 0,24, 48, and 72 hours. Cells pellets collected at indicated time points were resuspended in 100 ul of RIPA protein extraction buffer supplemented with protease inhibitor cocktail and placed on ice for 30 minutes. Samples were then centrifuged at 12.000 g for 10 minutes at 4°C and the supernatant was collected. Protein content was evaluated through Bradford assay, using Pierce™ Coomassie Plus (Thermo Scientific). Coomassie was diluted 1:1 with water and 1ml of the solution was placed in a semi-micro cuvette along with 5ul of sample. The absorbance was read at 595 nm in a spectrophotometer (Cary 60 UV-VIS, Agilent Technologies). Protein concentration was determined using bovine serum albumin as a standard protein."

Metabolite extraction

1. The computational part stated the uptake rates are based on “time 0h and 48h” (line 583 for example). Here it states, that fresh medium was added after 18h…. Does that mean, that 0h is actually 18h for the computational side, and did they know/was that taken into account? I assume, that that medium was used, as nothing else is stated for the medium analysis?

We have better clarified in that we consider as time 0 the time at which the medium is added (i.e. 18 hours after plating).

2. Sidenote for future experiments: Quenching with liquid nitrogen instead of the -80 freezer?

We thank the reviewer for the valuable suggestion. We will try in the future a comparison between the two quenching methods.

3. 70% power for the sonicater does not provide any insight if the model is not mentioned.

The sonicator is a HD 2070 from Bandelin Sonoplus equipped with a MS73 probe. We have included these details in the revised manuscript..

4. centrifugal vacuum concentrator speed, pressure?

We have included in the revised manuscript information about these not adjustable parameters of the concentrator that we also report below.
We used the V-AQ function, suitable for aqueous solutions. The speed is not adjustable (248 x g) as well as the pressure (pump maintains an ultimate pressure of at least 20 mbar. Its suction capacity is at least 1.8 m3/h).

LC MS metabolic profiling

1. LC gradients are strange, as they 0 min A, 2 min A, 4 min 0.99A, 10 min 0.98 A… why is the 2 min step there? Similar for 11 and 15 min, as both is 0.7 A. The 5 min post-run is with A? So total runtime per sample 21 minutes?

PFP column can be used for selective analysis of compounds such as polar compounds that contain hydroxyl, carboxyl, nitro or other polar groups when using highly aqueous mobile phases—including 100% water. Therefore the method was optimized using two min in isocratic they are able to allow a better separation of the polar compounds that otherwise could coelute. Total run time per sample is 21 minutes, but the 5 minutes post-run are not acquired.

We included this information in the revised manuscrip**t.**

2. Which gases did you use?

The Agilent Jet Stream ESI-MS source uses super-heated nitrogen as a gas.

We included this information in the revised manuscript.

Metabolite quantification

1. With this information I cannot reproduce the experiments. This also indicates, that only the 48h samples were analyzed and not the 0h? Where they sterile filtered? How much was used? …

We revised the Metabolites quantification in the media samples section in Materials and Methods including more information. Now it's:
"Absolute quantification of glucose, lactate, glutamine, and glutamate in fresh medium at t=0 and in spent media after 48 hours of growth was determined enzymatically using YSI2950 bioanalyzer (YSI
Incorporated, Yellow Springs, OH, USA). Media collected from experiments were thawed and centrifuged at 2000 rpm for 5 minutes prior to analysis. 1ml of sample is placed in a 1.5 ml tube and a volume of 25 ul was injected for the analysis. YSI bioanalyzer employed enzyme-based biosensors for measuring glucose, lactate, glutamate and glutamine concentrations. The biosensors used oxidases-containing membranes for oxidizing substrates, releasing hydrogen peroxide. The hydrogen peroxide is detected amperometrically at a platinum electrode surface. The current flow at the electrode is directly proportional to the hydrogen peroxide concentration and hence to the substrate concentration. Glucose, lactate, glutamine and glutamate standard solutions were used to calibrate the instrument.

RNA extraction

1. How did the authors get from "8 x 106 cells" to "each pellet"?

2. Line 738 "RNase-free" vs line 373 "RNAse-free"

3. "after purification" How? Miniprep? Details.

We extended the RNA extraction section as follows:

"Total RNA was extracted from at least 8 x 106 cells by using RNeasy Mini Kit (Qiagen), according to manufacturer's protocol. In detail, cells were disrupt by using lysis buffer, homogenized and, finally, resuspended by adding 30 L of RNase-free water. Following RNA isolation, DNAse treatment was performed using DNAse I, RNase-free (ThermoFisher Scientific). After purification by ethanol precipitation, to assess the final RNA yield and purity, the spectrophotometer NanoDrop ND-1000 (NanoDrop Technologies) was employed by the means of 260/280 and 260/230 ratios. The 2200 TapeStation instrument (Agilent 741Technologies) also evaluated the RNA quality, in order to assess the RNA Integrity Number Equivalent (RIN) for each processed sample."

RNA-Seq

1. This paragraph needs a work-through, as there are quite many "the" missing, and some sentences are long and thus hard to read.
2. Line 746 "Starting form each RNA isolated" –> isolate; "as first" at first
3. Line 751 "diluted at" diluted to

Writing of this section was improved according to reviewer' suggestions. Reference to deposited raw data was also included.

Raw reads are available in NCBI Short Reads Archive (SRA) under Accession Number under Accession Number PRJNA767228".

Link to data for reviewers:

https://dataview.ncbi.nlm.nih.gov/object/PRJNA767228?reviewer=34rlhb5fn5sddehh4s4ot2pno8. The deposited project will be made public after acceptance

General material and methods:

The language and understandability is mostly good. It is possible to follow the descriptions. There are multiple citations missing and I am not able to reproduce the presented methods/work based on the description.
The link (NOT provided in materials and methods) to the github (https://github.com/qLSLab/integrate) is not working (potentially a private repository?), and thus an evaluation of the proposed pipeline and the methods is also not possible.
In addition the data generated in the experimental parts is missing to a large extend, and none of the data in the supplementary files is linked in the methods part.

As detailed above we have now made the github repository public and properly referenced data in the supplementary material and to raw data deposited in public databases.
We have included missing citations in the revised manuscript.

Introduction

General note: The authors are inconsistent: In lines 42++ the examples are S1 and E1 with/without italics, with/without subscript, with/without []. Are they different? Another examples are constraint based vs constraint-based, metabolomics data vs metabolomic data.

Thank you for the comments. We have now made the annotations and formulas consistent throughout the paper.

1. "In recent years, the biological role of metabolism has been reconsidered" as first sentence is quite a statement without any literature sources. Please do add citations.
2. The pattern of missing citations for statements made continues here. I will not further highlight them, but assume, that the authors find and fix these issues themselves.
3. The authors should also consider, for better readability, to shorten sentences. For example, lines 21 to 30 (10 lines) are two sentences. There are quite a few 5-line-sentences. Also adding paragraphs would increase readability (e.g. there is

none the first 35 lines). For example I had to read the intro two times to realize, that lines 30 to 35 are the foundation for the manuscript. I feel this should be more highlighted by elements such as paragraphs, but also more than a “it is worth noting”.

4. The sentence “The gene expression layer sets the upper bound for any given flux” should be clarified.

We extensively rewrote and rearranged the structure of the whole Introduction to improve its readability, reducing the length of the sentences, restructuring paragraph organization, adding a few references and taking into account specific suggestions by the reviewer. We believe that the new introduction is easier to read and better conveys our arguments and reasoning.

5. The example of the two cells A and B and the metabolic reaction could be illustrated.

Thanks for the suggestion. We provided an accompanying image in the Supplementary Materials that we copy below for reviewer's convenience.

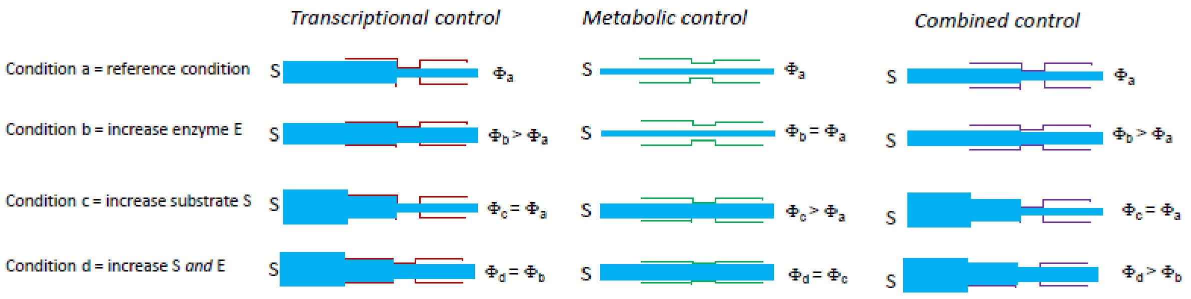

6. I suggest a change “S1 is in excess, so the flux through E1 is independent” to “the flux through r1”

This was indeed a misspell. We completely rearranged this paragraph and provided an accompanying image in the Supplementary Materials.

7. Lines 61++ (“Yet, the integration of these […]”): There has been multiple integrations of these type of omics data, specifically transciptomics data (e.g. gxFBA; for review see e.g. https://dx.plos.org/10.1371/journal.pcbi.1003580). The authors should clarify that specifically the integration of BOTH datasets is discussed here

With “integration of different omics data” we actually meant integration with one another, we did not mention integration with a genome-wide model in this paragraph. A few lines below on the contrary we did discuss integration of transcriptomics data into genome-wide models and cited the review indicated by the reviewer. We tried to disambiguate the two concepts in the revised Introduction section.

8. “As a proof-of-principle […]” Please also include the results of the pipeline-integration, how does it predict in comparison to the lab measurements that the authors generated?

We have now anticipated in the Introduction Section that INTEGRATE proved able to identify statistically relevant co-variations between experimental metabolomics and computationally predicted fluxomics,

General note: Please expand the literature review on transcriptomics and metabolomics integration into genome-scale metabolic models, specifically the well-established methods/approaches. Compare these to the proposed parts here. The whole experimental part is mentioned in half a sentence, the authors should expand on that and potentially also link that more to the start of the introduction/give potential use cases this pipeline could impact science and society.

The aim of our work is not proposing a new method to integrate transcriptomics data into metabolic models. For this reason we limited ourselves to citing exhaustive review articles on this topic, rather than listing the plethora of state of the art methods, although some more recent, original research articles have been cited in the revised manuscript. We have instead cited and briefly described all (to our knowledge) main state of the art methods about integration of metabolomics WITH transcriptomics data, which is our aim. We have extended the discussion on the comparison with the state art in the revised Introduction section. A brief sentence on the possible translational relevance of the class of approaches has been inserted and the subject is more extensively discussed in the introduction.

Results

The INTEGRATE pipeline

1. Lines 108++: I think, the authors should add/clarify, what the omics data is: Is it a single measurement of a single cell line (I like the definition in the start of the Results part), or is it measurements from two starts and taking the “delta” of these? From the output of INTEGRATE I’d assume, it’s the latter, but I feel this should be clarified.

We have now specified (in that paragraph) that transcriptomics and metabolomics data are cross-sectional and that extracellular fluxes are at steady state. The way in which strady extracellular-fluxes are estimated does not affect the workflow, hence we do not explain how we calculate them in this section.

2. I was actually looking in the Methods for “the method”, but since the Pipeline is a result as well, I guess, it is fine to have a detailed description here, discussing the different steps in detail. Unfortunately, the description is only scratching on the surface of the pipelines capabilities, for a suggestion see point 6 here.

We wanted to be concise in the results section and to avoid details that do not represent the core of the proposed pipeline and that can be modified discretionally, such as the choice of the specific statistical tests or of the concordance measure. Yet, we have now largely modified and extended the methods section to make it more independent from the Results

section. As suggested by the reviewer, we have also included a toy model example illustrating all the steps to obtain the concordance scores, including RAS and RPS computation in Supplementary Figure S4.

3. Reactions missing a metabolite measured my metabolomics are omitted. What is about the Transcriptomics then? They are omitted as well?

Our transcriptomics dataset fully covers the genes included in our metabolic network. The problem of missing genes is quite rare in bulk RNA-seq.

4. Figure 1) RNA-seq read counts = transcriptomics?! Indicate which of the input datasets is optional.

As we did not try to run the pipeline without extracellular fluxes, we decided to remove any reference to the distinction between optional and mandatory inputs throughout the (revised) paper. The principle of constraint modeling is that the more constraints one has the more one can reduce the solution space. Of course if one does not have extracellular fluxes he/she can still run the pipeline with less constraints.

Maybe differentiate “Input, Pipeline, and Output” a little better? The model is also an input, right? And the derived Datasets are an output? I assume, since I cannot test the pipeline code, that the derived datasets are saved output, and the variation concordance analysis is an additional output?

Thanks for the suggestion. We modified the labels to better distinguish inputs from outputs, as follows: “input datasets”, “input metabolic model”, “intermediate outputs”, “final output”.

The figure description could be expanded a little.

We expanded the caption.

5. Table 1) Caption below the table? Maybe include ER and PR abbreviations?

We expanded the description of the table in the caption.

6. Line 134: “Once the three reaction-oriented datasets are obtained,[…]” I am still not sure, how that works exactly. Some short example toy-code could be nice to highlight some of the core features of the proposed pipeline.

As mentioned above, we included a diagram with a toy model example illustrating all the steps of the pipeline in Supplementary Figure S4.

7. Line 137 “a proper statistical test” The authors should either mention the test here, or link to a later description in the manuscript, potentially give examples they used to clarify what a “proper” statistical test is.

We have now linked the Material and Methods section for the specific tests used in this study. Yet other standard statistical tests might alternatively be used.

8. Note that the description of the proposed pipeline, which is the main result of the manuscript (as the cell tests are a test case), is merely one page of text. I feel the functionality of the pipeline and more detailed functionality deserves some more focus.

As a general rule, the Results and Materials and Methods should be clearly differentiated. By way of example, San Francisco Edit, a company specializing in revision of scientific manuscripts, provides guidelines for writing an effective Materials and Methods section, from which we extract some key points reported below.
"In the Materials and Methods section you explain clearly how you conducted your study in order to: (1) enable readers to evaluate the work performed and (2) permit others to replicate your study. …. The main consideration is to ensure that enough detail is provided to verify your findings and to enable the replication of the study.
You should maintain a balance between brevity (you cannot describe every technical issue) and completeness (you need to give adequate detail so that readers know what happened)."
This differentiation is more straightforward for papers that simply apply standard methods, than for papers that propose and apply new methods. We did our best to make the two sections as independent as possible (especially in the revised version). Yet they cannot be completely stand-alone, otherwise they would simply be two copies of the same section. For this reason, we reported technical details in the Methods section, and novel intuitions/ideas/assumptions in the Results section.

Selected breast cancer cell lines […]

1. Fig 2) Lines around the plot. Figure quality (especially D), Red and green in a figure, Alignment (A, C, and D are shifted.

We improved the quality of the Figure and used a new, more colorblind-friendly palette.

2. Here is a link to "all the experimental data". Is it possible to add units? And a description of what is what? I am missing the (growth) data, the Figure 2 is made from.

We have included a "read me" in the first spreadsheet of the file explaining the data contained in the various sheets. We have included the growth data.

The ENGRO2 model

1. The model is based on RECON3D, as the authors state, basically a curated core model of the RECON3D. Can the authors include a table with the comparing key parameters of the 3 models (recon, Engro1/2) such as number of reactions, genes,… and potentially also specifically the number of reactions within their core model, that they added/curated in comparison to the recon3/Enrgo1/HMR?

We inserted the required information in the main text, as follows:
"The final version of the ENGRO2 core model consists of 496 reactions, 422 metabolites and 494 genes. As a comparison, we report that these values are respectively 84, 67, 216 for ENGRO1, and 13543, 8399, 3697 for Recon3D"

2. I would have liked to see the explanation of lines 211-216 in materials and methods, as this actually explains the authors general model reconstruction steps. I suggest moving these lines, as they are actually materials and methods (or a discussion of the model/results for that matter) but not results.

We thank the reviewer for the advice. Accordingly, we moved this part in the Methods section.

That said, I think almost the whole chapter should be Discussion as the authors thoroughly discuss the model generation and the reasoning for it. However, the authors do not present the model per sé, as for example I suggested in the previous comment: How many reactions does the model have? Genes, GPRs, and other model typical parameters.

As mentioned above, we included all these details in the revised manuscript.

3. Side note: "Recon3D" or RECON3D?... PLEASE be consistent!

We have standardized the notation.

Cell relative metabolic models

1. I do not agree with the "Constraints on nutrient availability" as more available nutrient will not necessarily increase the uptake. For example, an Ecoli cannot take up more than 16mmol/gCDW glucose even if the amount of glucose would be increased 10-fold. For lower concentrations, up to the limit this is reasonable, however. Hence, I suggest that the authors include a cap for what is biologically possible/plausible, and potentially lower the upper bond of the other cell instead?

The point raised by the reviewer is correct. Yet, unfortunately, this kind of information is not available in humo sapiens. Because it is not possible to remain 'agnostic' in constraint-based modeling, nutrients must be constrained somehow. Many studies leave all nutrient constraints equally bound to 1000. Yet this would imply stating that the intake rate of, for instance an essential amino acid can be equal to that of oxygen and glucose. In our experience, this will lead to even less realistic results than assuming that the ratio between glucose utilization and isoleucine utilization reflect (at most) the ratio of their concentration in the medium. For this reason we apply the same approximation also for the same metabolite across different cell lines, as done in other studies.

Moreover, we verified that for the case of glutamine, for which we have data and which is the metabolite that mainly differ across the two medium used it is reasonable to assume a monotonic correlation between metabolite concentration and metabolite consumption (concentration at time 48h - concentration at time 0), as we show in the plot below.

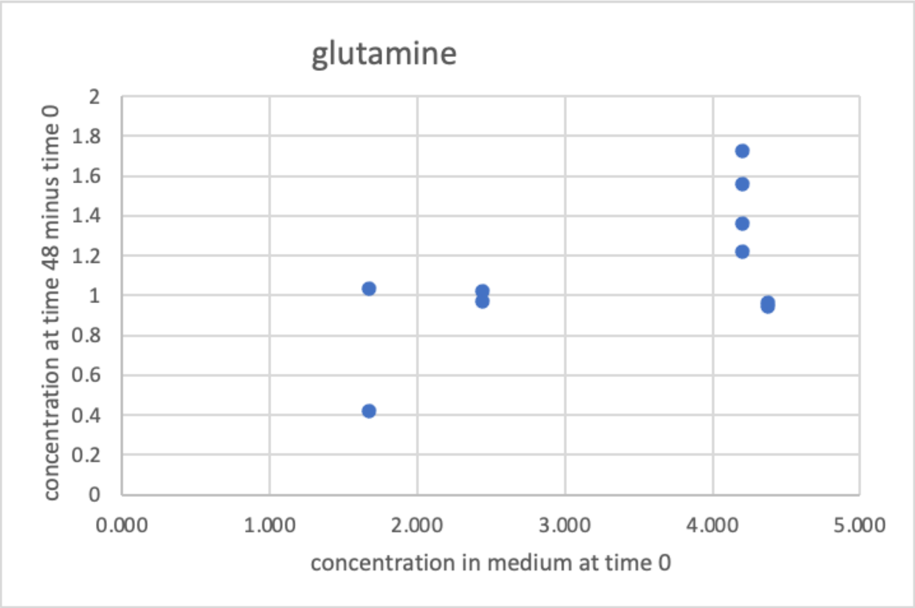

2. Lines 279-283 are discussion, no results.

Perhaps it was not clear that we were explaining how we generated the plots in Figure 3. We rephrased the lines to better clarify this point in the revised manuscript, while also better describing the different combinations of constraints in Figure 3.

3. Line 284: “In Fig. 3 it is evident […]” The authors should be careful with these statements, as it might not be evident for every reader.

We agree. We have removed these statements.

Further, the “iterative application of the 3 constraints” -> Which of the methods did the authors apply iteratively, and how many times? That as well as the implementation should also be mentioned in materials and methods.

The description was actually misleading, as we are not iterating. We have now clarified both here and in Material and Methods (First paragraph of section titled “From ENGRO2 to cell-relative constraint-based models”) that the three constraints can be applied either separately or simultaneously. We have also clarified that even if the three types of constraints are incremental (meaning that type 2 cannot be applied after type 3) one can omit type 2 constraints and apply type 3 directly after type 1.

4. Fig 3: Red/green; “evaluation of the iterative […]” suggestion to include “how” – e.g. mention tSNE; The figure is 2 Panels with 4 subfigures each. The authors list “type 1” in the

description, and have a black box with a “1” in the figure. Suggestion to make that A1 A2, A3 maybe? Or add Boxes around A and B and use a headline for the figure, e.g. “nutrient change”, “extracellular fluxes”,.. to make it easier to understand; Why did the authors not include the metabolomics?; wet -> in vivo; What is growth yield?

We modified the figure to make each subfigure a different panel and with a less ambiguous title. We extended the explanation on how we computed the yield when commenting the Figure in the results section.

We remark that metabolomics is never used as a constraint in our pipeline. We better motivated this choice in the revised Introduction section. We report the paragraph below.

“Yet using metabolomics to predict fluxes requires a priori assumptions on the relationship between metabolites and fluxes. For example, iReMet-flux \cite{sajitz2016iremet} assumes linearity between substrate abundance and flux for all reactions. Pandey et al. \cite{pandey2019enhanced}, assume instead that the variation in the abundance of a given metabolite must translate into a variation in the fluxes responsible for either its production or consumption.
Our aim is instead to use metabolomics data to assess a posteriori whether a variation in a flux is consistent with a given assumption. Our novel assumption is that evidence for a monotonic relationship between variations in fluxes and variations in substrate abundances, and a concurrent non monotonic relationship between flux variation and and enzyme activity variation, indicate that a reactions is controlled metabolically”

5. Line 290 “decrease the distance” -> What kind of distance measure was used? I assume, that the authors mean a clearer separation in the plots, and they should maybe reformulate that.

Thank you for the suggestion. We reformulated the sentence.

6. Line 292: “growth yield” What do the authors mean? Growth rate? Number of cells? Cell Dry Weight production? How did the authors measure/calculate these (Move lines 293-297 to materials and methods). Can the authors potentially also have these values in a table?

We left the brief definitions in Results, since in the absence of a dedicated section in Material and Methods we think that the reader may benefit from having the explanation here, rather than hunting for the definitions elsewhere.

The rewritten paragraph reads as follows:

“The experimental growth yield (reported in Supplementary File 1) was computed for each of the two collected biological replicates as the ratio of the total proteins produced over 48 hours (grams/hour, determined by Bradford analysis) over the glucose consumed over the same period (grams/liter, obtained by YSI analysis of spent medium.} The counterpart *in silico* growth yield was computed as the median ratio of the protein synthesis flux over the glucose uptake flux values. Both computed fluxes were expressed as grams consumed or produced/hour”.

We included the experimental growth yield values in the Supplementary Material and below

7. Line 293 “wet and computational data” Please reformulate that…. I know what the authors mean, but “wet data” is a formulation that makes me smile a little, which we would also (and only) say to/about “wet-lab-people”.

The wording was indeed improper. We replaced “wet” with “experimental”.

8. So far the authors did “only” discuss transcriptomics and secretomics data with the use of the model. (I would consider the matching of experimental medium to the computational medium a normal modelling application) Why did the authors not include their metabolomics data and evaluate the full potential of INTEGRATE

As discussed a few lines above we intentionally did not include metabolomics data as constraints, but we compare fluxes against metabolomics data (in the form of RPSs) *a posteriori*. By doing so the RPS and FFD datasets are fully independent, otherwise we could not argue whether a reaction is metabolically controlled, which is the principal aim of our work. Integration of transcriptomics and secretomics data is functional to predict fluxes, we do not intend to put any emphasis on our specific method to integrate transcriptomics data, any other available method of choice can be used (in principle) in the pipeline for that specific task. We better clarified this point in the revised Introduction section.

INTEGRATE discriminates reactions […]

1. Here are again parts that are materials and methods (e.g. Cohen’s Kappa coefficient) as well as discussion.

We agree with the reviewer that this explanation belongs more to Materials and Methods section. Nevertheless, we prefer to leave it there because explaining how to interpret Cohen’s Kappa values is fundamental to understanding results. In this way even readers less mathematically oriented should be able to follow our workflow without excessive jumping from one section to another. As outlined above, in methodological papers, the choice between what to include in Results and in Materials and Methods is somehow discretional. We strived to provide a balanced format: inevitably we have to accept that people with different backgrounds would make different choices.

2. Fig. 4: red/green (and that in combination with grey) Alignment of the plots, as well as the quality. Placement of the color bars, only description of one of them. “cohen”, “ACONT” – These require a description what they are. Remove the sentence-repetition.

We improved figure coloring and labels and alignments. We better describe labels in the caption. Yet, the color bars were already positioned in the correct corresponding panel

3. Can the authors include a table with the concordance values and the numbers of reactions that score with the respective values?

We included in the revised manuscript a Q-Q (fig 4C) to compare the distribution of concordance scores that we obtained against their empirical distribution (i.e. distribution that one would obtain by chance) that should address the reviewer comment.

For reviewers' convenience we report a histogram of the concordance scores below.

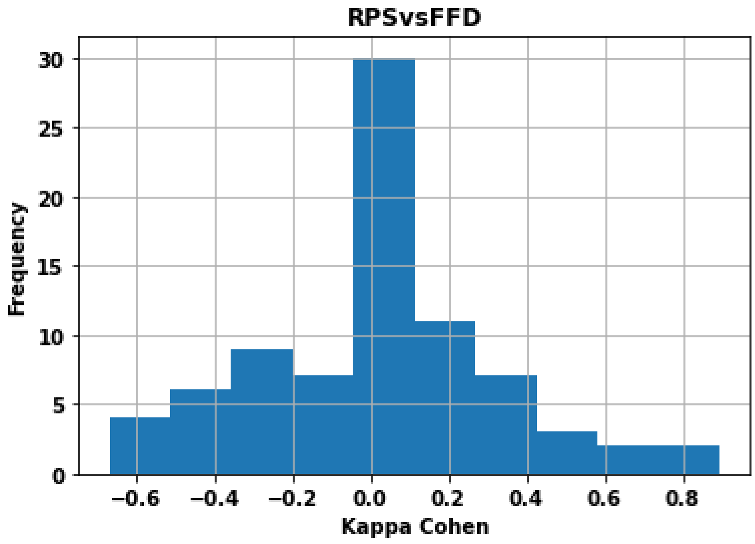

A few fluxes well […]

1. “The ACONT reaction, which we previously discussed”; The discussion continues in line 366. I suggest moving these points to the discussion and keep to the results in the results section.

We removed this section according to reviewer 1 suggestions.

2. Fig. 5 A box around the figure; Flux value [unit]; RPS/FDD black, ACONT/OCOAT1m grey; “according to the flux of cytosolic ACONT” -> reformulate; 5 -> five; “C) Same as B” I would repeat the description, but I guess due to the figure size, that is fine.

The caption has been rephrased.

3. Line 371: suggest to remove “in Figure 5”

We removed the expression from the sentence.

4. Line 373+ “similar to what was done in 32” Add a brief description into materials and methods. If it only was “similar” and not the same way, please state the differences so the work it is reproducible.

The differences are in the details and mainly have to do with the visualization and with the fact that we are representing many sampled fluxes instead of the optimal distribution. Nevertheless, We removed this figure and relative section according to reviewer 1 suggestions.

5. 389 “only in the extent” -> to the extent
We corrected the typo

6. Fig. 6: red/green; please us the real amino acid names, not the model metabolite IDs; Why not integrate the AA name into the y-Axis description? For most of them, the orange one (MCF102A) is hardly visible. Is it possible to highlight it somehow?;

We removed this figure and relative section according to reviewer 1 suggestions.

The figure description is vastly different from the previous ones, please be consistent. I think, it is a good idea to give a general explanation , but I think, the AAs should be mentioned earlier.

We removed this figure and relative section according to reviewer 1 suggestions.

7. Side question: Did the authors change the biomass? Since the contribution of the AAs to the biomass does not change, if the authors did not change the biomass coefficient. Hence, the contribution to biomass would directly correlate to the growth of the cells. The plotted uptake of AA and directly correlates with the growth (I assume, the biomass coefficient for the specific AAs were not variable) and I assume what is visible with the 1M samples is basically the flux variation in that correlation. Please do keep during the discussion (lines 380-387) in mind, that the models assume steady-state and mass conservation.

The biomass coefficient is indeed fixed and the contribution to biomass directly correlates with the growth rate, but we want to explore whether it directly correlates with the intake flux of the aminoacid. The y-axis label was actually misleading and we replaced it with “XXX flux drain to growth (mmol/gDW/h)?”. where XXX is the amino acid name. Yet, as already mentioned, we removed this figure and relative section according to reviewer 1 suggestions.

General comment results:

The authors mix results, materials and methods, and discussion for many parts. The result part should be a listing of results, which then are discussed in the discussion, if these are different chapters. I wonder why the authors did not evaluate all parts of INTEGRATE, as

they do not appear to evaluate the impact of the generated metabolomics data and thus loose the advantage over other extended transcriptomics integrations. The authors also do not show a direct evaluation of the pipeline, e.g. is it
better then other pipelines/easy to use methods, what is their advantage, how does it correlate to experiments?

As detailed in the point-by-point reply above, we better explained why we do not use the metabolomics data to set constraints and better compared our approach with state of the art in the revised Introduction.
We substantially re-written the Methods, Results and Discussion sections to better differentiate them.

Discussion
Lines 392-421 feel like a repetition of the previous results parts, whereas 422-427, 446-449,464-470 feel like a conclusion.

We streamlined and improved readability of the Discussion section and we combined Results and Discussion one Results/Discussion section."
