# Supplementary material for: INTEGRATE: Model-based multi-omics data integration to characterize multi-level metabolic regulation
Source: PLoS Comput Biol. 2022 Feb 7;18(2):e1009337. doi: 10.1371/journal.pcbi.1009337 (PMC8853556; doi:10.1371/journal.pcbi.1009337)
Supplement: Attachment — Submitted filename: ReplyMinor.pdf [file pcbi.1009337.s014.pdf]

Milan, January 11th 2022

Dear Editors and Reviewers,

please find enclosed the second revision of our manuscript entitled "*INTEGRATE: Model-based multi-omics data integration to characterize multi-level metabolic regulation*", submitted to **PloS Computational Biology**.

We are very glad that both reviewers appreciated the overall effort that we made to address their criticism and that they are now satisfied with our revised manuscript.

We addressed all the minor comments raised by reviewers, as detailed in the following. For your convenience, any changed and new text is marked in red, but we also provide an unmarked version of the manuscript.

We look forward to the publication of our manuscript in PloS Computational Biology.

Kind regards,
Chiara Damiani,
also on behalf of the other authors.

# Reviewer 1

Reviewer #1: I'm happy to see that the authors have made a true effort to address most of the shortcomings that were suggested previously, and I believe that the manuscript has improved a lot. The minor revisions requested below are mostly text or figure edits that in my opinion will improve the manuscript.

We thank the reviewer for the appreciation of our effort, for his/her constructive criticism, and for the minor comments that allowed us to further improve our manuscript.

Comments (line numbers refer to manuscript with changes higlighted)

- Line 64: Do you mean "a significant increase the abundance of enzyme E"? An increase in activity of enzyme E is by definition an increased flux the reaction r (Enzyme activity = moles of substrate converted per unit time).

We were referring to the theoretical activity of the enzyme, either caused by a variation in its abundance or k_cat. The theoretical activity does not correspond with the actual flux, which instead depends on the availability of substrate. Yet, we agree with the reviewer that this concept was misleading. For the sake of simplicity, and given that we are considering gene expression data, we modified the text by simply referring to variations in enzyme abundances.

- Line 77: .. when the amount of enzyme E increases.

We have now specified throughout the Introduction section that it is the amount of enzyme that increases.

- I think the examples of control mechanisms (line 76-86) can be more accurately explained, possibly by illustratin how the Michelis-Menten equation can be approximated when substrate concentration is << or >> than Km. -

We better illustrated the Michelis-Menten equation and clarified the examples of control mechanisms in the context of the equation.

Line 79: The description of metabolic control doesn't make sense. Increased enzyme and substrate abundance have to increase flux. I think it is more correct to say the rate is mostly affected by substrate concentration. If you consider the Michelis-Menten equation, enzyme abundance also affects reaction rate when S<

We thank the reviewer for this comment. We rewrote the paragraph to better clarify this concept.

- Line 123-124: This sentence should be rephrased.

- Line 133: typo: starting form

- Line 153: typo: A a proof ..

We corrected all the misspellings.

- Figure 2D: Maybe I'm misinterpreting the meaning of the size of each dot, but in my understanding it is the ratio of samples for each cell line for each metabolite above the average value for that metabolite across all cell lines. If this is correct, how can all the cell lines have large (close to 100%) of samples above the mean for some metabolites?

We apologize for the oversight. When changing the representation from Z-score to scaled average, the meaning of the dots' size switched from "fraction above the mean", to "fraction above zero" and we did not modify the caption accordingly. Yet, we believe that the former representation is more meaningful when dealing with metabolomics data. Hence, we decided to switch back to the Z-score representation, clarifying the interpretation of the plot both in the legend and in the caption.

- Figure 2F: Y-axis numbers too small

The font size was increased.

- Line 244-245: Seems like a word is missing here

We rephrased the sentence.

- Fig 3: Please report p-values for the calculated correlations

We thank the reviewer for the suggestion. We have now reported the p-values in the plot title.

- Line 425: missing parenthesis

We corrected the typo.

- Figure 5A, the two heatmaps miss titles (which is RPS and which is FFD)

We included the missing titles.

- Figure 5: A proper legend for colors/cell lines would be nice

We included an appropriate legend to the Figure.

- GitHub repository should be deposited at a permanent repository (e.g. Zenodo) in agreement with FAIR principles.

We thank the reviewer for this useful suggestion. We have now deposited the repository in Zenodo under the following permanent identifier:

10.5281/zenodo.582450 (https://zenodo.org/record/5824505#.YdbcXhPMLep).

# Reviewer 2

I would like to thank the authors for their contribution. I hope that they agree that the comments from me and the other Reviewer increased the readibility and value of the scientific contribution to the field.

I think there are still some minor improvements that could be done (language, sentence length,...) but over all I think the manuscript is good for publication.

We are very glad that the reviewer appreciated the overall effort to address her/his criticism. We do agree with the reviewer that the comments from both reviewers allowed us to largely improve our contribution.
We have carefully checked the final text, by also using the tool Grammarly. We corrected a large number of misspellings and streamlined many verbose sentences. We did not consider it necessary to highlight all such minor changes in the text.
